# Metagenomic Insights into the Uncultured Diversity and Physiology of Microbes in Four Hypersaline Soda Lake Brines

**DOI:** 10.3389/fmicb.2016.00211

**Published:** 2016-02-25

**Authors:** Charlotte D. Vavourakis, Rohit Ghai, Francisco Rodriguez-Valera, Dimitry Y. Sorokin, Susannah G. Tringe, Philip Hugenholtz, Gerard Muyzer

**Affiliations:** ^1^Microbial Systems Ecology, Department of Aquatic Microbiology, Institute for Biodiversity and Ecosystem Dynamics, University of AmsterdamAmsterdam, Netherlands; ^2^Evolutionary Genomics Group, Departamento de Producción Vegetal y Microbiología, Universidad Miguel HernándezSan Juan de Alicante, Spain; ^3^Department of Aquatic Microbial Ecology, Biology Centre of the Czech Academy of Sciences, Institute of HydrobiologyČeské Budějovice, Czech Republic; ^4^Research Centre of Biotechnology, Winogradsky Institute of Microbiology, Russian Academy of SciencesMoscow, Russia; ^5^Department of Biotechnology, Delft University of TechnologyDelft, Netherlands; ^6^The Department of Energy Joint Genome InstituteWalnut Creek, CA, USA; ^7^Australian Centre for Ecogenomics, School of Chemistry and Molecular Biosciences and Institute for Molecular Bioscience, The University of QueenslandBrisbane, QLD, Australia

**Keywords:** soda lake brines, *Nanohaloarchaea*, *Halobacteria*, *Bacteroidetes*, *hydrolytics*, cellulase, chitinase, rhodopsin

## Abstract

Soda lakes are salt lakes with a naturally alkaline pH due to evaporative concentration of sodium carbonates in the absence of major divalent cations. Hypersaline soda brines harbor microbial communities with a high species- and strain-level archaeal diversity and a large proportion of still uncultured poly-extremophiles compared to neutral brines of similar salinities. We present the first “metagenomic snapshots” of microbial communities thriving in the brines of four shallow soda lakes from the Kulunda Steppe (Altai, Russia) covering a salinity range from 170 to 400 g/L. Both amplicon sequencing of 16S rRNA fragments and direct metagenomic sequencing showed that the top-level taxa abundance was linked to the ambient salinity: *Bacteroidetes, Alpha*-, and *Gamma-proteobacteria* were dominant below a salinity of 250 g/L, *Euryarchaeota* at higher salinities. Within these taxa, amplicon sequences related to *Halorubrum, Natrinema, Gracilimonas*, purple non-sulfur bacteria (*Rhizobiales, Rhodobacter*, and *Rhodobaca*) and chemolithotrophic sulfur oxidizers (*Thioalkalivibrio*) were highly abundant. Twenty-four draft population genomes from novel members and ecotypes within the *Nanohaloarchaea, Halobacteria*, and *Bacteroidetes* were reconstructed to explore their metabolic features, environmental abundance and strategies for osmotic adaptation. The *Halobacteria*- and *Bacteroidetes*-related draft genomes belong to putative aerobic heterotrophs, likely with the capacity to ferment sugars in the absence of oxygen. Members from both taxonomic groups are likely involved in primary organic carbon degradation, since some of the reconstructed genomes encode the ability to hydrolyze recalcitrant substrates, such as cellulose and chitin. Putative sodium-pumping rhodopsins were found in both a *Flavobacteriaceae*- and a *Chitinophagaceae*-related draft genome. The predicted proteomes of both the latter and a *Rhodothermaceae*-related draft genome were indicative of a “salt-in” strategy of osmotic adaptation. The primary catabolic and respiratory pathways shared among all available reference genomes of *Nanohaloarchaea* and our novel genome reconstructions remain incomplete, but point to a primarily fermentative lifestyle. Encoded xenorhodopsins found in most drafts suggest that light plays an important role in the ecology of *Nanohaloarchaea*. Putative encoded halolysins and laccase-like oxidases might indicate the potential for extracellular degradation of proteins and peptides, and phenolic or aromatic compounds.

## Introduction

Modern soda lakes are characterized by high salt concentrations that can reach up to saturation and a stable high pH, typically between 9.5 and 11 (Jones et al., 1977; Tindall, 1988; Banciu and Sorokin, 2013). They occur in (semi-)arid regions world-wide where they form naturally through evaporative concentration. Their origin is not related to seawater (“athalassic”), but to seepage of groundwater that is rich in sodium ions (Na^+^) and CO_2_, but low in calcium (Ca^2+^), and magnesium (Mg^2+^) ions that would otherwise precipitate the dissolved carbonates. Whereas in the water column of “thalassic” environments and inland salt lakes, the dominant soluble salt is sodium chloride, sodium carbonate/bicarbonate salts dominate in soda lake brines and create a naturally buffered haloalkaline system. Moderately saline soda lakes are believed to be amongst the most productive aquatic environments on Earth (Melack, 1981). Complete biogeochemical carbon, nitrogen and sulfur cycles can still occur in hypersaline (salinity >5% w/v) soda lakes and become only partially hampered at salt-saturating conditions (Sorokin et al., 2014, 2015a, and references therein).

For hypersaline thalassic environments with circum-neutral pH, it is well-established from studies on solar salterns that salinity is the major environmental factor shaping planktonic prokaryotic community composition (e.g., Rodriguez-Valera et al., 1985; Oren, 1994; Casamayor et al., 2000, 2002; Benlloch et al., 2002; Ghai et al., 2011; Dillon et al., 2013). Seawater is led into an interconnected pond system where salinity gradually increases by evaporative concentration until sea salt can be harvested. Along the pond system, proteobacterial planktonic diversity decreases in favor of extreme halophilic *Archaea*, such as *Haloquadratum* sp. belonging to the class *Halobacteria* (phylum *Euryarchaeota*) and members of the candidate class *Nanohaloarchaea* (phylum *Nanohaloarchaeota*; Ghai et al., 2011; Narasingarao et al., 2012; Martínez-García et al., 2014), as well as some moderate and extreme halophilic *Bacteria* such as the *Gammaproteobacteria Spiribacter salinus* (López-Pérez et al., 2013; León et al., 2014), *Halovibrio* and *Halospina* (Sorokin et al., 2006), and *Salinibacter ruber* in the *Bacteroidetes* phylum (Antón et al., 2000, 2002; Mongodin et al., 2005).

A similar influence of prevailing salt concentrations on microbial community composition could be inferred from previous culture-independent studies of alkaline soda lake brines. Various bacterial genera belonging to the *Alphaproteobacteria, Firmicutes, Gammaproteobacteria, Bacteroidetes*, and *Cyanobacteria* are found in soda brines with salinities up to 250 g/L (Humayoun et al., 2003; Mesbah et al., 2007; Dimitriu et al., 2008; Pagaling et al., 2009; Asao et al., 2011; Lanzen et al., 2013), while above these salinities *Euryarchaeota* belonging to the *Halobacteria* and the *Methanosarcinales* are found to dominate in natural lakes (Grant et al., 1999; Ochsenreiter et al., 2002; Mesbah et al., 2007; reviewed by Sorokin et al., 2014, 2015a) and in artificial soda concentration ponds (Gareeb and Setati, 2009; Simachew et al., 2015).

Most culture-independent studies performed in these environments world-wide are in agreement that soda lakes harbor a surprisingly high 16S rRNA diversity and abundance of still uncharacterized microbes. At high salinities, the high diversity within several genera and species of the class *Halobacteria* is not in proportion to the relatively low number of soda lake isolates obtained from this group of mostly aerobic heterotrophs. Only recently the first alkaliphilic members of the *Halobacteria* capable of growing on cellulose and chitin were isolated from various soda lakes, showing that these organisms can fulfill an environmental role as primary organic matter degraders (Sorokin et al., 2015d). It is well-established, and has received considerable interest from the biotechnology-industry, that many members of the *Halobacteria* are able to degrade proteins and peptides via extra-cellular proteases, also referred to as “halolysins” (De Castro et al., 2006). Almost all of these halolysins are subtilisin-like serine proteases (peptidase subfamily S8A) and quite a few are isolated, including from alkaliphilic members of the *Halobacteria*. Many members of the phylum *Bacteroidetes* are also able to consume biopolymers, such as chitin, cellulose, xylan and proteins, and are therefore often viewed as a key group for organic matter remineralization in the marine environment (e.g., Bauer et al., 2006). Interestingly, with the exception of *Natronoflexus pectinovorans* (phylum *Bacteroidetes*), polysaccharide-degrading *Bacteria* isolated from soda lakes belong mostly to the *Firmicutes* (genera *Bacillus, Clostridium*), *Actinobacteria* (genera *Cellulomonas* and *Dietzia*), *Gammaproteobacteria* (*Alkalimonas, Marinimicrobium*), and *Fibrobacteres* (genus *Chitinovibrio*; Sorokin et al., 2014, 2015a).

With the advent of next generation sequencing, direct metagenomic approaches have been successfully applied to extreme environments harboring relatively low-diversity communities. They have proven very useful to construct snapshots of the overall microbial community structure and to aid in the reconstruction of draft or even complete genomes of uncultured microorganisms (e.g., Tyson et al., 2004; Ghai et al., 2011; Narasingarao et al., 2012; Sharon and Banfield, 2013; Fernández et al., 2014a,b). Notably, nanohaloarchaeal 16S rRNA sequences were first recovered from the hypersaline soda lake Magadi in the east-African Rift Valley (Grant et al., 1999) and since then four near-complete genomes belonging to the “*Candidatus* Nanohaloarchaea” have been reconstructed from metagenomes of various hypersaline aquatic habitats (Ghai et al., 2011; Narasingarao et al., 2012; Martínez-García et al., 2014). Based on 16S rRNA phylogeny of sequences recovered from hypersaline and haloalkaline habitats worldwide, *Nanohaloarchaea* were first viewed as a deep-branching sister-group of the class *Halobacteria* within the phylum *Euryarchaeota* (Narasingarao et al., 2012; Oren, 2014). Based on the recent genome reconstructions however, a new candidate phylum “*Nanohaloarchaeota*” within a “DPANN” super-phylum was proposed (Rinke et al., 2013; Castelle et al., 2015), but this view remains controversial since the outcome of archaeal phylogenetic analyses appears to depend on the choice of marker genes and reference genomes, among other factors (Petitjean et al., 2015). In addition to the absence of cultured isolates from this class and their disputed phylogeny, the lifestyle and ecological function of *Nanohaloarchaea* remains enigmatic.

Here we determined for the first time the overall structure of microbial communities from the brines of three natural hypersaline soda lakes and a trona crystallizer located in the Kulunda Steppe (Altai, Russia), covering a salinity range between 170 and 400 g/L with a combination of both 16S rRNA amplicon and direct metagenome sequence analyses. In the past 15 years, these lakes have been intensively studied by classical microbiology approaches providing a good background for the interpretation of the metagenomics results. We reconstructed several draft genomes of yet uncultured populations within the phylum *Bacteroidetes*, class *Halobacteria*, and candidate class *Nanohaloarchaea* in order to explore their metabolic potential with respect to organic carbon remineralization in soda brines and other putative roles in biogeochemical cycling.

## Materials and methods

### Site description, sample collection and DNA extraction

The four sampled soda lakes are located in the southern part of the Kulunda Steppe, South-Western Siberia (Altai region), Russia. The exact soda lake locations, chemical parameters and general appearance of the brine at the time of sampling are given in Table 1. The eutrophic lakes Bitter-1 (B1), Tanatar trona crystallizer (Tc), and Tanatar-5 (T5) are part of the Bitter and Tanatar systems, respectively, and were formed on tertiary depositions of trona-cemented sands (Isachenko, 1951). The oligotrophic Picturesque Lake (PL) is located in the depression of the Salt Lake Steppe (sediments - gray kaolinite clays). All lakes are hypersaline, shallow ponds subjected to a very unstable water regime and with temperature fluctuations from +35 to −35°C (Foti et al., 2008). At the time of sampling the salt concentration ranged from 170 to 400 g/L sodium carbonates/chloride and a pH from 9.5 to 10.3 in different lakes.

**Table 1:** **General features of the sampled soda lakes, the obtained metagenomic raw sequence reads, and the assembled contigs**.

**Lake**	**Lake Tanatar-5 (T5)**	**Picturesque Lake (PL)**	**Lake Tanatar trona crystallizer (Tc)**	**Lake Bitter-1 (B1)**
Location	51.62 N 79.84 E	51.73 N 79.87 E	51.65 N 79.75 E	51.67 N 79.91 E
Sampling year	2010	2010	2011	2010
Salinity (g/L)	170	250	300	400
pH brine	9.9	9.5	9.9	10.2
Na_2_CO_3_ alkalinity (M)	0.8	1.1	4.0	2.0
Total carbonate alkalinity (M)	1.9	2.8	5.0	4.4
Brine appearance	Muddy	Muddy	Orange-red	Oily yellow
Metagenome ID	T5-Br10	PL-Br10	Tc-Br11	B1-Br10
SRA Accession	INGZ–SRA052017	INHF–SRA052008	INHA–SRA052024	INHB–SRA052018
Number of read pairs	206,674,382	352,665,522	191,945,135	411,685,143
Estimated mean coverage	140x	190x	129x	202x
Number of assembled contigs	636,016	1,140,049	458,620	482,919
Number of contigs ≥5 kb	19,350	25,098	9,426	15,551
GenBank Accession (contigs ≥5 kb)	LFIK00000000	LKMJ00000000	LFFM00000000	LFCJ00000000
Average contig length (b)	1,005	974	895	1,115
Maximum contig length (b)	204,877	192,059	134,932	155,378
Total assembly length (b)	639,293,147	1,110,081,880	410,404,879	538,272,225

*Raw sequence reads have been deposited to the NCBI Sequence Read Archive (SRA) and contigs larger than 5 kb at DDBJ/EMBL/GenBank*.

Brine samples were collected in sterile glass bottles in July 2010 and 2011 and prefiltered (Whatman “White Ribbon” paper filters) to remove suspended sediment particles and aggregates. Cells from 1 L brine were pelleted by high speed centrifugation (30 min at 13,000 rpm with a Sorvall SLC-1500 rotor) and resuspended in 2-4 M NaCl. Smaller volumes were finally pelleted (10 min at 20,817 × g) in 2 mL Eppendorf tubes and stored at −70°C until further processing. High molecular weight DNA was extracted with the classical phenol/chloroform method (Marmur, 1961) and isopropanol precipitation after resuspending the pellets in 10 mL 0.1 M Tris (pH 8)/10 mM EDTA and pretreatment with lysozyme, SDS, proteinase K, and RNase. The quality and quantity of the extracted DNA was checked by agarose gel electrophoresis using standard markers of known concentration.

### Amplicon sequencing and analysis

The V6-V8 region of 16S rRNA was amplified using primers 926F (5′- cct atc ccc tgt gtg cct tgg cag tct cag AAA CTY AAA KGA ATT GRC GG- 3′) and 1392R (5′ - cca tct cat ccc tgc gtg tct ccg act cag - < XXXXX> - ACG GGC GGT GTG TRC – 3′) universal to both *Archaea* and *Bacteria* (Tremblay et al., 2015). Primer sequences were modified by the addition of 454 A or B adapter sequences. In addition, the reverse primer included a 5 bp barcode for multiplexing of samples during sequencing. Twenty-microlitre PCR reactions were performed in duplicate and pooled to minimize PCR bias using 0.4 μl Advantage GC 2 Polymerase Mix (Advantage-2 GC PCR Kit, Clontech), 4 μl 5X GC PCR buffer, 2 μl 5 M GC Melt Solution, 0.4 μl 10 mM dNTP mix (MBI Fermentas), 1.0 μl of each 25 nM primer, and 10 ng sample DNA. The thermal cycler protocol was 95°C for 3 min, 25 cycles of 95°C for 30 s, 50°C for 45 s, and 68°C for 90 s, and a final 10 min extension at 68°C. PCR amplicons were purified using SPRI Beads (Beckman Coulter) and quantified using a Qubit fluorometer (Invitrogen). PCR products were diluted to 10 ng/μl and mixed in equal concentrations. Emulsion PCR and sequencing of the PCR amplicons were performed following the Roche 454 GS FLX Titanium technology manufacturer's instructions. Sequencing tags were analyzed using the software tools PyroTagger (http://pyrotagger.jgi-psf.org) and MacQIIME (http://www.wernerlab.org/software/).

### Metagenome sequencing and raw read analysis

Environmental DNA (500 ng) was sheared using the Covaris E210 and size selected for 270 bp fragments using Agencourt Ampure Beads (Beckman Coulter) prior to library construction with the KAPA Illumina library creation kit (KAPA Biosystems). Each library was quantified by qPCR and sequenced on one lane of the Illumina HiSeq 2000 platform to generate paired-end 150 bp reads. Low quality regions of the paired-end raw reads were trimmed using DynamicTrim (Cox et al., 2010). Top-level taxonomic profiles of the metagenomes were determined according to Ghai et al. (2011). Candidate 16S rRNA encoded metagenomic reads were initially selected by comparing a subsample of 2 million sequence pairs with a small manually-curated database. Reads with a minimum alignment length of 90 bases (min identity 80%, *e*-value 1e-5) were extracted and compared to sequences in the Ribosomal Database Project (Cole et al., 2014). The best-named hit was used to classify the reads to top level taxa. Chloroplast 16S rRNA was manually filtered out and taxa containing less than 1% of the total assigned 16S rRNA reads were not considered.

### Reconstruction and analysis of draft genomes

Assembly of the metagenomic reads was performed with MEGAHIT v1.0.1-13-gd6932f9 (Li et al., 2015). Protein coding genes on contigs longer than 5kb were predicted with Prodigal (Hyatt et al., 2010), tRNAs with tRNASCAN (Lowe and Eddy, 1997), rRNAs using meta_rna (Huang et al., 2009) and annotated using BLAST against the non-redundant database Pfam (Bateman et al., 2004), COGs (Tatusov et al., 2003), and TIGRfams (Haft et al., 2001). Genes at the end of contigs and incomplete genes without valid start/stop codons were removed. Based on sequence similarity against the non-redundant NCBI database, the best hit for each gene was determined and used to bin to top level taxa. Only those contigs that contained a minimum of three genes and had not more than half of the genes hitting different taxa were retained. Further binning of the contigs belonging to *Bacteroidetes, Nanohaloarchaea*, or *Halobacteria* was done by differential coverage binning with CONCOCT (Alneberg et al., 2014) after using subsets of 20 million sequence reads of each dataset to estimate coverage by mapping reads with min 95% nucleotide identity and 50 bp overlap. Possible bin contamination and strain heterogeneity were estimated with CheckM v0.9.7 (Parks et al., 2014) and only those with reasonable concatenated length (>0.5–0.8 Mb) and with contamination below 5% were retained for subsequent analysis.

Taxonomic affiliation of the selected bins was determined via phylogenetic trees based on concatenates of all proteins identified within Clusters of Orthologous Groups (COG; Tatusov et al., 2003) and shared with complete or near-complete reference genomes (RefSeq NCBI, plasmids excluded), since no 16S rRNA genes were found on the contigs. Shared proteins were concatenated and aligned using Kalign (Lassmann and Sonnhammer, 2005) and a maximum-likelihood tree was made using the software program MEGA version 6 (Tamura et al., 2013). The completeness of the reconstructed bacterial and archaeal genomes was assessed based on the presence of 112 essential genes (Albertsen et al., 2013) and 53 core genes (Narasingarao et al., 2012) respectively, using HMMER. Using an *e*-value cut-off of 1e-5, lower and upper bound predictions of completeness were obtained using all predicted genes or only the best annotations (hcov and qcov >80%). Average nucleotide identity (ANI) and conserved DNA fraction between reconstructed and/or reference genomes were calculated based on the whole genome sequence as described by Goris et al. (2007).

The carbon-, nitrogen- and sulfur metabolic potential of the draft genomes was inferred from all proteins identified within COG, as well as from KEGG pathways and BRITE hierarchies after re-annotating the predicted proteins from the standard draft genomes automatically using KAAS (KEGG Automatic Annotation Server: http://www.genome.jp/kegg/kaas/; Moriya et al., 2007). Additionally, assigned K-numbers were used to screen for the presence of functional marker genes (Lauro et al., 2011; Llorens-Marès et al., 2015). Putative encoded carbohydrate-active enzymes in the draft genomes were identified by sequenced-based annotation of the predicted proteins from all coding sequences using the CAZymes Analysis Toolkit (CAT; Park et al., 2010; Lombard et al., 2014).

To determine the general mode of osmotic adaptation of the putative microorganisms for which we reconstructed the genomes, the isoelectric points (pI) of the coding sequences with a minimum length of 30 amino acids were calculated using the program IEP in the EMBOSS package (Rice et al., 2000). The pI distribution (bin width 0.1) for each composite genome was compared to the pI distribution of selected, complete reference genomes available in RefSeq. Genes likely to be involved in osmotic adaptation were identified through their annotations and best-hits (see above), namely those involved in osmolyte synthesis and uptake or those encoding putative ion transporters and channels.

### Read recruitments

Population abundance in the different soda lake brines was estimated via read recruitments to the draft genomes from subsamples of 20 million reads from the different metagenomic datasets. A cut-off of 95% identity in at least 50 bases was used and the number of hits was normalized against the size of the genomes and metagenomes and expressed as Reads Per Kilobase of sequenced reads per Gigabase of mapped reads (RPKG; López-Pérez et al., 2013).

### Data availability

The raw sequence reads of the four metagenomics datasets have been deposited to the NCBI Sequence Read Archive (SRA; Accession Numbers given in Table 1). The assembled contigs larger than 5 kb have been deposited as Whole Genome Shotgun projects at DDBJ/EMBL/GenBank (Accession Numbers given in Table 1). The reconstructed draft genomes have been deposited as Whole Genome Shotgun projects at DDBJ/EMBL/GenBank under the accessions LKMK00000000-LKNH00000000 (complete list given in Supplementary Table 2). All versions described in this paper are version XXXX01000000.

## Results and discussion

### Microbial community composition based on 16S rRNA gene sequences

#### Top level taxa abundance in relation to salinity

**Figure 1:**
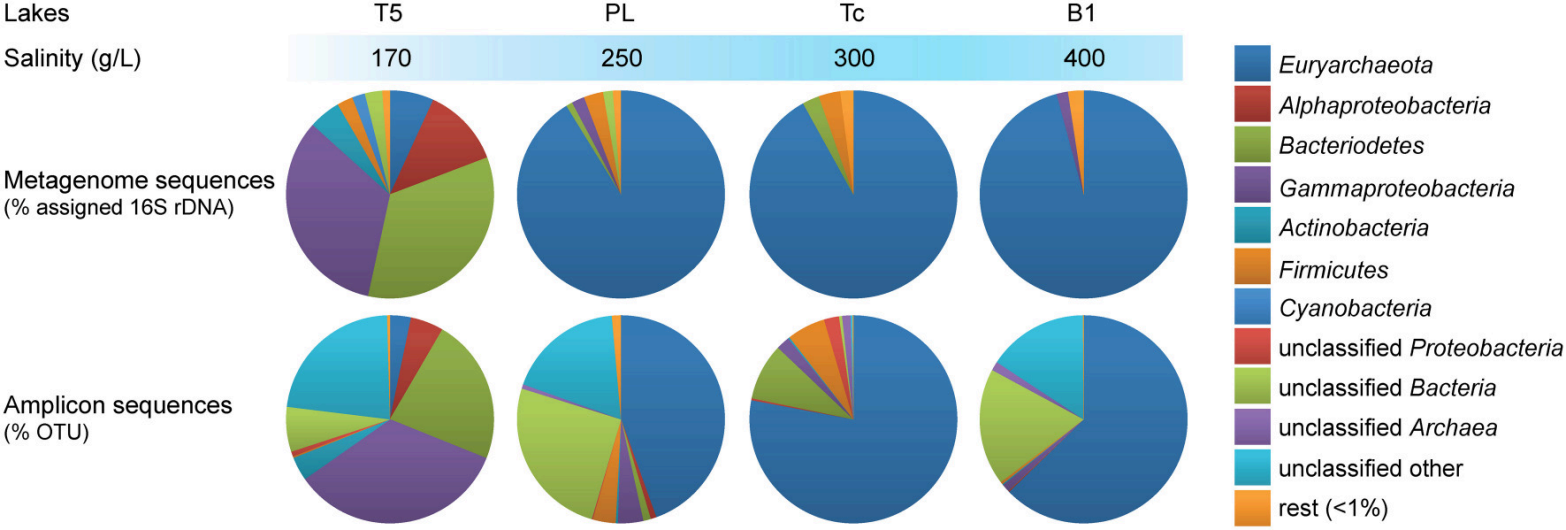
**Taxonomic profiles of the microbiota from hypersaline soda lake brines**. **Top**: distribution of sampled metagenomic reads encoding fragments of 16S rRNA genes assigned to top level taxa. **Bottom**: distribution of the OTUs assigned to the amplicon sequences. T5, Tanatar-5; PL, Picturesque Lake; Tc, Tanatar trona crystallizer; B1, Bitter-1.

Microbial community composition of the different soda lake brines was compared based on metagenomic reads containing fragments of the 16S rRNA gene as well as on OTUs assigned to the amplicon sequences (Figure 1). The majority of the selected metagenomic reads originating from the saturated salt brines (total salinity ≥250 g/L) of Bitter-1 (B1), Tanatar trona crystallizer (Tc), and Picturesque Lake (PL) belonged to the archaeal phylum *Euryarchaeota* (>91%). Those from Tanatar-5 (T5) with a total salinity of 170 g/L belonged to the bacterial taxa *Bacteroidetes* (34%), *Gammaproteobacteria* (33%), and *Alphaproteobacteria* (12%) and to a lesser extent to the *Actinobacteria, Firmicutes, Cyanobacteria*, and unclassified bacterial groups (< 5%). *Euryarchaeota* still constituted a considerable fraction of the prokaryotic community in the brine of T5 (7%). Similarly, 16S rRNA gene amplicon sequences belonging to *Euryarchaeota* dominated in the three most saline brine datasets, while those belonging to *Bacteria* dominated the lower salinity dataset. Our results are in agreement with previous culture-independent studies that reported the dominance of *Euryarchaeota* in 16S rRNA gene clone libraries from hypersaline soda lakes (Grant et al., 1999; Ochsenreiter et al., 2002; Mesbah et al., 2007; Simachew et al., 2015). Moreover, all bacterial top level taxa that were previously found to be common in soda lakes world-wide with a maximum salinity of 250 g/L (Humayoun et al., 2003; Mesbah et al., 2007; Dimitriu et al., 2008; Pagaling et al., 2009; Asao et al., 2011; Lanzen et al., 2013) appeared abundant (>1%) in both our metagenomic and amplicon T5 dataset.

Amplicon and shotgun sequence-based taxonomic profiling introduced different biases. While the direct metagenomic approach might have left rare taxa undetected (Lynch and Neufeld, 2015), amplification biases in complex DNA mixtures might have distorted the original 16S rRNA gene ratios. Most evidently for the three most saline lakes, rare (here < 1%) bacterial taxa were overrepresented in the amplicon datasets compared to the metagenomes, while archaeal 16S sequences were underrepresented. Furthermore, between 16 and 23% of the 16S amplicon sequences from the brines of B1, PL and T5 could not be assigned to any top level bacterial or archaeal taxon (“unclassified”). Since no “unclassified” sequences were found in taxonomic profiles derived from the metagenomes, these are suspected to originate from eukaryote organelles other than chloroplasts. *Cyanobacteria* were absent in the three most saline brines when considering the directly sequenced metagenomes, but present in the same brines when considering the amplicon sequences. Finally, on the phylum and class level, the total number of detected taxa (including “Unclassified” *Bacteria* and *Archaea*) in the metagenomes was inversely related to salinity with six taxa detected for B1 (400 g/L salt) and 14 for T5 (Supplementary Table 3). This trend was not apparent in the amplicon sequencing datasets where the brines of PL and T5 appeared the most and the least rich (19 and 11 top level taxa OTUs), respectively.

#### Cultured vs. unknown members of the most abundant top level taxa

While the directly sequenced, short-read metagenomes yielded in our opinion more realistic snapshots of the relative 16S rRNA gene abundances of the high-level community constituents, we considered only the longer amplicon sequences (~450 nt vs. < 150 nt) for further phylogenetic assignment down to the genus-level, focusing on the identified abundant top-level taxa (Table 2).

**Table 2:** **Genus assignments of the detected OTUs from 16S rRNA amplicon sequences belonging to the most abundant top level taxa from different soda lake brines**.

**% Assigned of total OTUs**	**B1**	**Tc**	**PL**	**T5**
***Euryarchaeota***				
*Halobacteriales*^a^*/Halobacteriaceae*^a^*/Halorubrum*	23.5	23.7	5.8	0.00
*Halobacteriales*^b^*/Halobacteriaceae*^b^*/Natrinema*	0.94	1.69	0.35	0.00
*Halobacteriales*^c^*/Halobacteriaceae*^c^/other^d^	0.11	0.69	0.59	0.00
*Halobacteriales*^c^*/Halobacteriaceae*^c^*/*none	38.2	51.7	37.7	3.2
*Methanosarcinales*/*Methanosarcinaceae*/*Methanolobus*	0.00	0.00	0.00	0.04
Other^e^^,^ ^f^	0.03	0.20	0.03	0.00
***Bacteroidetes***				
*Bacteroidales*/*Marinilabiaceae*/none				
*Flavobacteriales*/*Flavobacteriaceae*/none	0.00	0.00	0.01	1.05
*Flavobacteriales*/other^g^	0.00	0.00	0.01	0.00
*Sphingobacteriales*/*Chitinophagaceae*/*Balneola*	0.01	0.00	0.00	0.00
*Sphingobacteriales*/*Chitinophagaceae*/*Gracilimonas*	0.02	8.3	0.72	18.8
*Sphingobacteriales*/*Chitinophagaceae*/none	0.00	0.22	0.25	2.50
*Sphingobacteriales*/other^g^^,^ ^i^	0.00	0.00	0.004	0.00
*Sphingobacteriales*/*Rhodothermaceae*/none	0.00	0.24	0.00	0.00
*Sphingobacteriales*/*Rhodothermaceae*/*Salisaeta*	0.00	0.06	0.00	0.00
Other^h^^,^ ^j^	0.01	0.01	0.18	0.24
***Alphaproteobacteria***				
*Rhizobiales*/other^g^^,^ ^k^	0.00	0.02	0.21	1.65
*Rhodobacterales*/*Rhodobacteraceae*/*Rhodobaca*	0.00	0.00	0.00	0.04
*Rhodobacterales*/*Rhodobacteraceae*/*Rhodobacter*	0.00	0.00	0.01	0.28
*Rhodobacterales*/*Rhodobacteraceae*/*Roseinatronobacter*	0.01	0.00	0.09	1.13
*Rhodobacterales/Rhodobacteraceae/none*	0.14	0.28	0.45	1.81
*Rhodospirillales*/other^g^	0.00	0.00	0.01	0.00
*Rhodospirillales*/*Rhodospirillaceae*/none	0.00	0.00	0.05	0.24
Other^h^	0.00	0.00	0.07	0.00
***Gammaproteobacteria***				
*Alteromonadales*/*Alteromonadaceae*/*Haliela*	0.01	0.00	0.00	0.00
*Alteromonadales*/*Idiomarinaceae*/none	0.01	0.00	0.01	0.00
*Alteromonadales*/other^g^	0.01	0.00	0.02	0.12
*Chromatiales*/*Chromatiaceae*/*Halochromatium*	0.00	0.00	0.00	0.04
*Chromatiales*/*Ectothiorhodospiraceae*/*Thioalkalivibrio*	0.07	0.26	0.71	12.0
*Chromatiales*/*Ectothiorhodospiraceae*/*Aquisalimonas*	0.01	0.01	0.13	0.00
*Chromatiales*/*Ectothiorhodospiraceae*/*Halorhodospira*	0.15	0.57	0.15	0.04
*Chromatiales*/*Ectothiorhodospiraceae*/other^l^	0.00	0.00	0.02	0.00
*Chromatiales*/*Ectothiorhodospiraceae*/none	0.00	0.01	0.01	0.16
*Chromatiales*/*Halothiobacillaceae*/*Thioalkalibacter*	0.00	0.00	0.01	0.00
*Chromatiales*/other^g^	0.00	0.00	0.01	0.40
*Gammapr. incertae sedis*/*Methylonatrum*/none	0.00	0.00	0.01	0.00
*Legionellales*/*Legionellaceae*/none	0.00	0.00	0.002	0.00
*Oceanospirillales*/*Hahellaceae*/*Hahella*	0.003	0.00	0.00	0.00
*Oceanospirillales*/*Halomonadaceae*/*Halomonas*	0.86	0.01	0.57	0.60
*Oceanospirillales*/*Halomonadaceae*/none	0.00	0.03	0.81	0.00
*Oceanospirillales*/*Halomonadaceae*/*Salicola*	0.00	0.00	0.002	0.00
*Oceanospirillales*/other^g^	0.00	0.00	0.06	0.56
*Thiotrichales*/*Piscirickettsiaceae*/*Thioalkalimicrobium*	0.00	0.00	0.004	0.00
*Xanthomonadales*/*Xanthomonadaceae*/none	0.00	0.004	0.00	0.00
*Xanthomonadales*/*Xanthomonadaceae*/*Stenotrophomonas*	0.00	0.00	0.002	0.00
Other^h^	0.09	1.22	1.41	20.25
**Remaining OTUs**	**35.9**	**10.8**	**49.5**	**34.7**

*T5, Tanatar-5; PL, Picturesque Lake; Tc, Tanatar trona crystallizer; B1, Bitter-1*.

A *Haloferacales/Haloferacaceae acc. to Gupta et al. (2014)*.

B *Natrialbales/Natrialbaceae according to Gupta et al. (2014)*.

C *Classification before Gupta et al. (2014)*.

D *Natronorubrum, Halopiger, Haloplanus, Halostagnicola, Haloterrigena, Natrialba, Natronococcus, Natronolimnobius, Natronomonas*.

E *Thermoplasmatales*.

F *No class assigned*.

G *No family assigned*.

H *No order assigned*.

I *Family Saprospiraceae*.

J *Order Bacteroidales*.

K *Family Bradyrhizobiaceae*.

L *Alkalilimnicola, Thioalkalispira, Thiohalospira*.

##### Euryarchaeota

More than 99% of the OTUs assigned to *Euryarchaeota* in the three amplicon sequencing datasets from the three most saline brines of PL, Tc, and B1, were affiliated with 11 distinct and known genera from the class *Halobacteria*. Among them, *Halorubrum*- and *Natrinema*-related sequences were the most abundant ones. The remaining largest fraction of the OTUs, as well as all *Halobacteria*-related sequences from the brine of T5, could not be assigned to any known genus.

Few *Methanosarcinaceae*-related sequences of the genus *Methanolobus* (class “*Methanomicrobia”*) were found in the brine of T5 (1.2% of *Euryarchaeota*-related OTUs or 0.04% of the total OTUs—see Table 2). The presence of this specific genus of moderately salt-tolerant, methylotrophic methanogens was also detected in 16S clone libraries obtained from the hypersaline lake Fazda (Wadi an Natron, Egypt; Mesbah et al., 2007). More recently, *Methanolobus* was also found in the sediments of hypersaline soda lakes of the Kulunda Steppe by a functional gene assay (Sorokin et al., 2015b). In addition, two *Methanolobus* isolates were obtained and potential methanogenic activity up to soda saturating conditions confirmed. Although methanogens are typically sediment-affiliated and suspended clay particles might have passed the pre-filtration step in this study, oxygen solubility in hypersaline brines is low (e.g., Grant, 2004) and therefore *Methanolobus*-related sequences might also originate from methanogens thriving in the brine of T5 under anoxic conditions.

##### Bacteroidetes

Most of the *Bacteroidetes* OTUs in T5 belonged to the genus *Gracilimonas* (~83%). This genus comprises a group of marine (Choi et al., 2009; Cho et al., 2013) and salt mine (Wang et al., 2013) isolates and related 16S gene fragments were recovered recently from a haloalkaline soil (Keshri et al., 2013). In the datasets of the more saline brines of B1, Tc, and PL, *Gracilimonas*-related sequences were also the dominant *Bacteroidetes*. The two important remaining *Bacteroidetes* fractions in the brine of T5 were affiliated with unknown genera from the families *Chitinophagaceae* (~11%) and *Flavobacteriaceae* (~5%).

##### Alphaproteobacteria

From the OTUs assigned to the *Alphaproteobacteria* in the T5 dataset, the dominant fraction (~63%) was affiliated with the family *Rhodobacteraceae* (order *Rhodobacterales*) and belonged to the genera *Roseinonatronobacter, Rhodobacter* and *Rhodobaca*. The genus *Roseinonatronobacter* comprises the haloalkaliphilic members of a unique group of aerobic, bacteriochlorophyll *a*-containing, sulfur-oxidizing lithoheterotrophs that were first isolated from soda lake microbial mats (Sorokin et al., 2000) and later from the low-salt epilimnion of the alkaline Mono Lake (Boldareva et al., 2007). Haloalkaliphilic members of the purple non-sulfur bacteria belonging to the latter two genera are frequently detected in soda lakes (Rees et al., 2004; Kompantseva et al., 2007; Asao et al., 2011). While members of the genus *Rhodobaca* were isolated from soda lakes (Milford et al., 2000; Boldareva et al., 2008), so far no alkaliphilic *Rhodobacter* isolates are obtained. Most of the *Rhodobacteraceae*-related OTUs from T5 could not be assigned to a known genus, which was also a conclusion from previous research on Kulunda soda lakes that focused on this group (Kompantseva et al., 2010). Another large alphaproteobacterial fraction (~32%) was assigned to the order *Rhizobiales*, but no family could be assigned. The presence of the rhizobial genus *Mesorhizobium* was reported previously in the sediment and dry mud from a Kenyan soda lake (Mwirichia et al., 2011) and in a recent molecular survey of Kulunda soda lake sediments (Tourova et al., 2014). Although there is a high probability that the *Rhizobiales*-related sequences in this study are from terrestrial origin, the order includes, besides plant rhizobia, plant- and animal-pathogens, photosynthetic purple non-sulfur bacteria that occur in soda lakes, such as the genera *Rhodopseudomonas, Rhodoblastus*, and *Rhodomicrobium* (Tourova et al., 2011). In the datasets of the more moderate saline brines of PL and T5, a substantial fraction of the alphaproteobacterial OTUs affiliated with the *Rhodospirillaceae*, another phylogenetic group of purple non-sulfur bacteria (Imhoff, 1995), still without soda lake isolates.

##### Gammaproteobacteria

From the OTUs assigned to the *Gammaproteobacteria* in the T5 dataset, the dominant fraction (~59%) was affiliated with no known order; the largest remaining fractions belonged to the genera *Thioalkalivibrio* (order *Chromatiales*; ~37%) and *Halomonas* (order *Oceanospirillales*; ~2%), comprising halo-alkaliphilic sulfur-oxidizing bacteria and aerobic heterotrophs commonly occurring in soda lakes with various salinities (Sorokin et al., 2014). In contrast, from the OTUs assigned to the *Gammaproteobacteria* in the dataset B1, the soda lake with the highest salinity, the dominant fractions belonged to *Halomonas* (~71%) and purple sulfur bacteria (genus *Halorhodospira*, order *Chromatiales*; ~13%).

### Draft genomes of putative predominant, yet still uncultured community members

**Figure 2:**
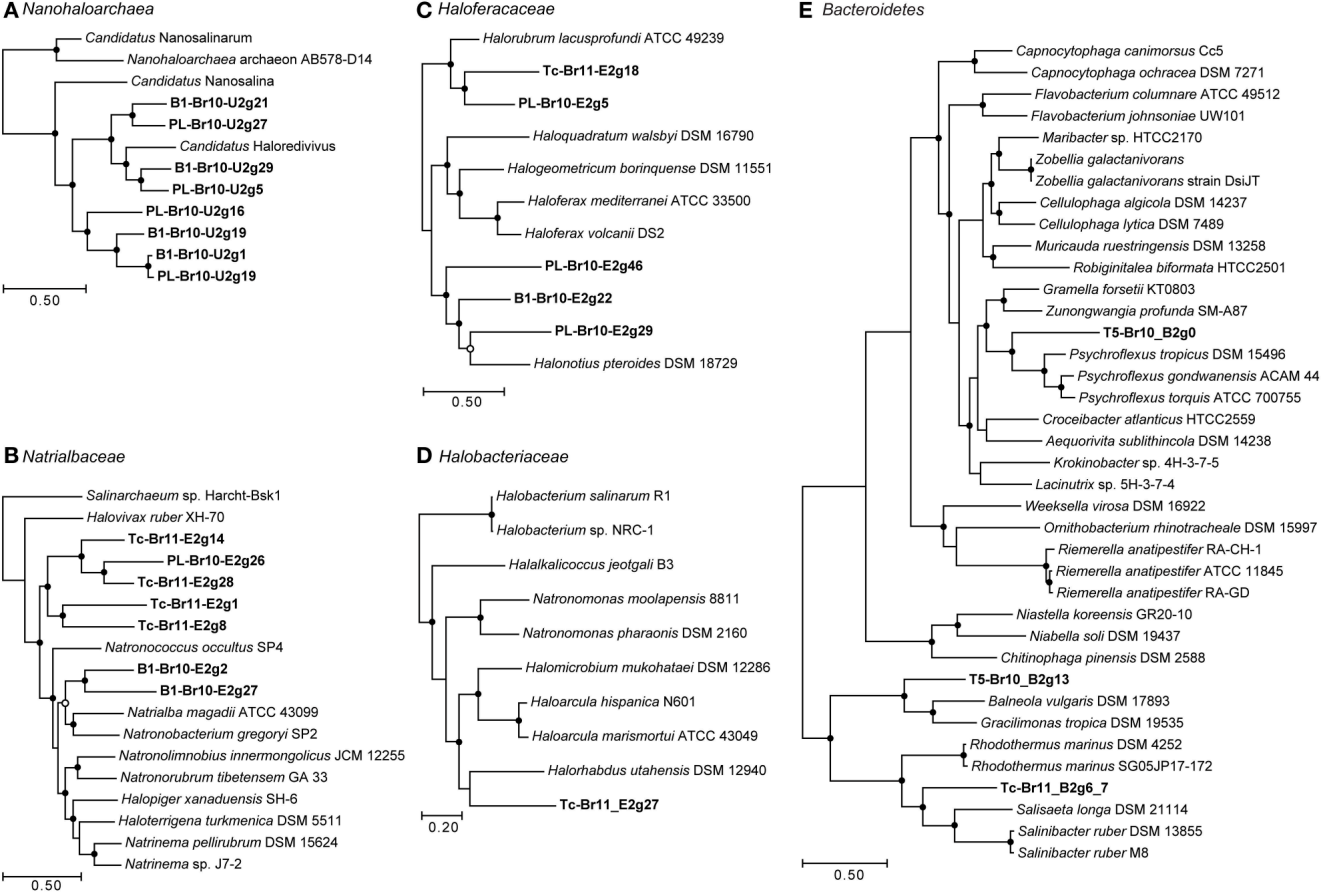
**Maximum-likelihood phylogenetic trees based on shared concatenated COGs between draft genomes reconstructed from the metagenomes and (near-)complete reference genomes**. Filled circles at the nodes represent bootstrap values (100x) of 90–100%, open circles of 80–90%. Bars indicate the amount of sequence difference. All outgroups were pruned from the trees. **(A)** 62 COGs shared between reconstructed draft genomes and reference genomes from *Candidatus* “Nanohaloarchaea.” Outgroup = *Halobacteria*; **(B)** 142 COGs shared between reconstructed draft genomes and reference genomes from the family *Natrialbaceae* (class *Halobacteria*). Outgroup = *Archaeoglobi*; **(C)** 104 COGs shared between reconstructed draft genomes and reference genomes from the family *Haloferacaceae* (class *Halobacteria*). Outgroup = *Archaeoglobi*; **(D)** 427 COGs shared between the reconstructed genome Tc-Br11_E2g27 and reference genomes from the family *Halobacteriaceae* (class *Halobacteria*). Outgroup = *Archaeoglobi*; **(E)** 228 COGs shared between the *Bacteroidetes*-related reconstructs and genomes from *Bacteroidetes Order II insertae sedis* (family *Rhodothermaceae*), *Chitinophagaceae*, and *Flavobacteriaceae*. Outgroup = *Aquificae*.

**Figure 3:**
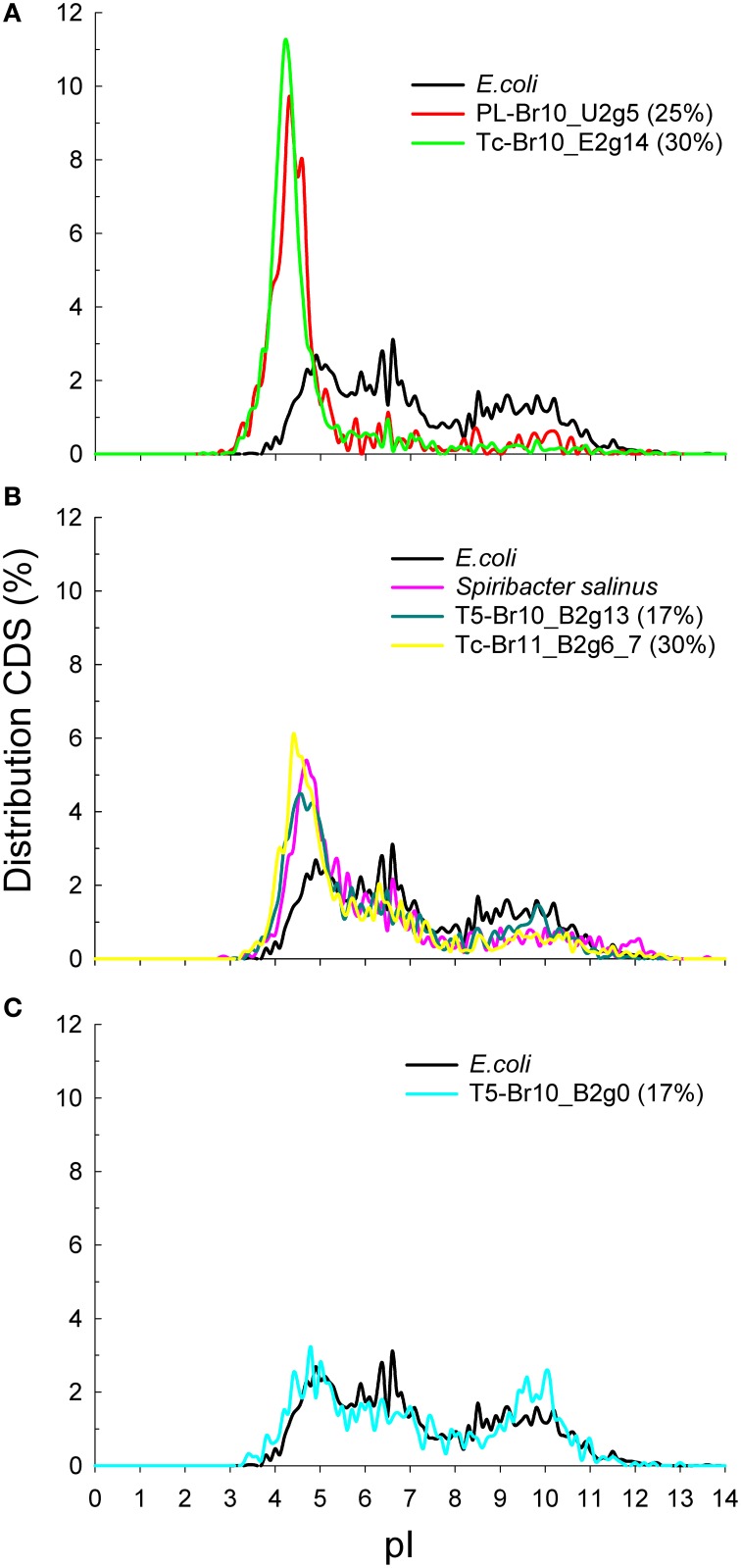
**Predicted isoelectric points (pI) for the coding sequences (CDS) of a selection of draft genomes and complete reference genomes indicative for different strategies of osmotic adaptation. (A)** archaeal “salt-in”: all archaeal draft genomes had a similar pI-profile, only one *Nanohaloarchaea*- and one *Natrialbaceae*-related draft genome are shown here; **(B)** bacterial “salt-in”; **(C)** “salt-out.” The salinities of the brines from which the draft genomes were reconstructed are depicted in between brackets (in %).

After assembling the metagenomic reads into contigs and classifying all ≥5 kb contigs to top-level taxa, we obtained several high-quality (< 4% estimated contamination using CheckM) draft genomes of uncultured members of the *Euryarchaeota, Bacteroidetes* and the candidate class *Nanohaloarchaea* (Table 3), as well as a large number of contigs belonging to *Eukaryota, Gamma*- and *Alphaproteobacteria* and *Actinobacteria* (Supplementary Table 1). The obtained draft genomes represent “population” genomes i.e., not clonal (Sharon and Banfield, 2013) of single species or strains. Below we discuss their inferred phylogeny based on genome trees (Figures 2A–E) and average nucleotide identities (ANIs; Supplementary Tables 4–8) with known organisms, their metabolic potential and principal modes of osmotic adaptation (Figure 3).

**Table 3:** **Phylogenetic affiliation and general features of selected reconstructed draft genomes**.

**Name < Id>**	**C**	**L**	**GC%**	**ORF**	**tRNA**	**16S**	**5S**	**23S**	**Cont**	**Str het**	**Compl**
*Chitinophagaceae* bacterium T5-Br10_B2g13	258	4.30	41	7004	35	0	2	0	2.1	0.0	80.2–91.9
*Flavobacteriaceae* bacterium T5-Br10_B2g0	132	1.49	33	2494	21	0	0	0	0.9	33.3	61.3-71.2
*Rhodothermaceae* bacterium Tc-Br11_B2g6_7	109	3.20	62	5220	38	1	1	1	1.1	0.0	79.3-92.8
*Halobacteriaceae* archaeon Tc-Br11_E2g27	205	2.10	55	3920	25	0	0	0	0.4	0.0	69.3–79.2
*Haloferacaceae* archaeon PL-Br10_E2g46	171	1.44	51	2586	19	0	0	0	0.5	25.0	67.9–77.4
*Haloferacaceae* archaeon B1-Br10_E2g22	152	2.37	58	4738	35	0	1	0	3.2	35.7	73.6–83.0
*Haloferacaceae* archaeon PL-Br10_E2g29	134	1.28	62	2478	21	0	0	0	1.6	44.4	62.3–73.6
*Halorubrum* sp. PL-Br10_E2g5	160	1.48	65	2756	19	0	0	0	0.4	50.0	79.2-83.0
*Haloferacaceae* archaeon Tc-Br11_E2g18	139	1.27	67	2372	15	0	0	0	0.0	0.0	62.3–75.5
*Nanohaloarchaea* archaeon B1-Br10_U2g1	26	0.710	43	1634	29	0	0	1	0.0	0.0	84.9–94.3
*Nanohaloarchaea* archaeon B1-Br10_U2g21	24	0.816	39	1756	34	0	1	1	2.8	50.0	81.1–86.8
*Nanohaloarchaea* archaeon B1-Br10_U2g19	35	0.663	41	1472	19	1	0	0	0.0	0.0	81.1–90.6
*Nanohaloarchaea* archaeon B1-Br10_U2g29	35	0.526	40	1182	19	0	1	0	1.0	0.0	64.2–73.6
*Nanohaloarchaea* archaeon PL-Br10_U2g5	26	0.84	41	1936	34	1	1	1	0.0	0.0	88.7–94.3
*Nanohaloarchaea* archaeon PL-Br10_U2g19	55	0.76	42	1702	34	0	2	2	0.9	0.0	81.1–90.6
*Nanohaloarchaea* archaeon PL-Br10_U2g16	54	0.65	42	1448	18	0	0	0	2.3	20.0	81.1–90.6
*Nanohaloarchaea* archaeon PL-Br10_U2g27	52	0.58	43	1290	22	3	0	2	0.9	0.0	73.6–77.4
*Natrialbaceae* archaeon B1-Br10_E2g2	251	2.875	66	5368	30	0	4	0	3.7	11.8	79.2–84.9
*Natrialbaceae* archaeon B1-Br10_E2g27	207	2.01	60	3710	22	0	1	0	0.8	0.0	62.3–71.7
*Natrialbaceae* archaeon Tc-Br11_E2g1	210	2.16	65	3990	27	0	0	0	1.8	20.0	75.5–84.9
*Natrialbaceae* archaeon Tc-Br11_E2g14	136	3.16	63	5916	48	0	0	0	1.6	0.0	84.7–94.3
*Natrialbaceae* archaeon Tc-Br11_E2g28	216	2.33	61	4342	34	0	2	1	0.3	0.0	71.7–86.8
*Natrialbaceae* archaeon Tc-Br11_E2g8	192	2.29	69	4206	32	0	1	0	0.7	16.7	71.7–83.0
*Natrialbaceae* archaeon PL-Br10_E2g26	167	1.46	61	2646	18	0	2	0	0.0	0.0	67.9–81.1

*C, # contigs; L, length in Mb; GC%, the average GC content; ORF, # open reading frames (CDS); tRNA, # tRNA sequences; 16S, 5S, and 23S = resp. # rRNA; Cont and Str het = % of bin contamination and strain heterogeneity estimated with CheckM; Compl = lower and upper bounds of predictions of % completeness based on 53 archaeal core genes or 111 single copy genes for the bacterial drafts*.

#### Candidate class *Nanohaloarchaea*

##### Phylogeny

From B1-Br10 and PL-Br10 we obtained eight draft genomes belonging to low-GC Archaea of the candidate class *Nanohaloarchaea* (Table 3; Figure 2A; Supplementary Table 4). Based on the genome tree, four of these reconstructions, two from each brine, were most closely related to the draft genome of “*Candidatus* Haloredivivus” obtained from metagenomes in a solar saltern pond (Ghai et al., 2011). They belonged to four different genera based on average nucleotide identity (all ANIs < 74.9%). The remaining four draft genomes belonged to one to three different genera, distantly related to “*Candidatus* Nanosalina” obtained from a hypersaline lake (Narasingarao et al., 2012; all ANIs < 74.5%), while the drafts B1-Br10-U2g1 and PL-Br10-U2g19 belonged to the same species thriving at different brines (ANI = 99.4%).

##### Xenorhodopsins and heterotrophy

We found several genes that support the predominant heterotrophic lifestyle previously proposed for “*Candidatus* Haloredivivus” (Ghai et al., 2011; Narasingarao et al., 2012). Notably, out of the eight draft genomes, six encoded putative xenorhodopsins for which the exact physiological role still remains enigmatic (Ugalde et al., 2011; Supplementary Figure 1, Supplementary Table 9). Starch-degrading metabolism was evident from encoded alpha-amylases and related glucoside hydrolases (CAZy families GH13 and GH57) found in seven out of eight drafts. In five drafts subtilisin-like proteins (peptidase S8) were encoded, suggesting degradation potential for extracellular proteins and peptides. Furthermore, in B1-Br10_U2g21 and B1-Br10_U2g19 we found laccase-like Cu-oxidases encoded that may be involved in the breakdown of phenolic compounds, ring cleavage of aromatic compounds or lignin (Ausec et al., 2011). A near-complete Embden-Meyerhoff pathway was encoded in all drafts, which include some typical archaeal enzymes such as an ADP-dependent phosphofructokinase/glucokinase (EC 2.7.1.147) and a 2,3-bisphosphoglycerate-independent phosphoglycerate mutase (EC 5.4.2.12; Siebers and Schönheit, 2005). The draft genomes also encoded a “classical” glucose-6-phosphate isomerase as found in most *Eukarya* and *Bacteria*. Moreover, they encoded an eukaryal-type Class I fructose-1,6-biphosphate aldolase (EC 4.1.2.13), which is unusual in *Archaea*, only shared with some members of the *Halobacteria* and *Nanoarchaeota* (Siebers et al., 2001; Say and Fuchs, 2010). The potential for conversion of pyruvate to acetyl CoA was encoded by a pyruvate dehydrogenase complex, while a pyruvate:ferredoxin oxidoreductase (pyruvate synthase; EC 1.2.7.1) was only encoded in the reference genome of “*Candidatus* Nanosalinarum.”

##### Fermentative lifestyle

Several genes related to central carbon metabolism and energy production were present in our draft genomes and the four publicly available drafts of *Nanohaloarchaea* described by Ghai et al. (2011), Narasingarao et al. (2012), and Martínez-García et al. (2014) suggest an anaerobic fermentative lifestyle, which contrasts to the principally aerobic respiratory metabolism suggested previously for “*Candidatus* Nanosalina” and “*Candidatus* Nanosalinarum” by Narasingarao et al. (2012). First, we found an ADP-forming acetyl-CoA synthase (EC 6.2.1.13), typical for acetate-forming anaerobic fermentative *Archaea* and facultative anaerobic *Halobacteria* (Siebers and Schönheit, 2005) as well as a malic enzyme (EC 1.1.1.38/39), a lactate dehydrogenase (the latter was also reported by Narasingarao et al., 2012), ferredoxin and a pyruvate:ferrodoxin oxidoreductase which suggests at least the potential for incomplete fermentation. Second, while most draft genomes encoded a H^+^-translocating NADH:ubiquinone reductase (EC 1.6.5.3), a plastocyanin-like type I copper protein (most likely involved in electron transfer reactions, e.g., Martin-Cuadrado et al., 2015) and V-type ATPases, none contained identifiable aerobic terminal oxidases. Andrade et al. (2015) most recently reported the absence of cytochrome *c* oxidases in novel *Nanohaloarchaea*-related draft genomes as well. Finally, in contrast to Narasingarao et al. (2012) we did not find a complete or reduced TCA-cycle in any of the available drafts, only a putative citrate synthase (EC 2.3.3.1) and both subunits of a putative succinyl CoA synthetase (EC 6.2.1.5), as well as a putative citryl CoA ligase (EC 4.1.3.34). In the available genome of “*Candidatus* Haloredivivus” however, the succinyl CoA synthetase was annotated as a citryl-CoA synthetase and had 35% sequence identity (Blast-P; *e*-value 4e-66) to the citryl-CoA synthetase of *Hydrogenobacter thermophilus* (bacterium phylum *Aquificae*). Citryl-CoA synthetase has been shown to act in the latter organism in concert with citryl-CoA lyase as an alternative citrate cleavage pathway to the reductive TCA cycle (Hügler et al., 2007). Hypothetically for the *Nanohaloarchaea*, both enzymes, together with malic enzyme, could perhaps function in the ATP-dependent regeneration of acetyl-CoA and oxaloacetate rather than in the aerobic TCA cycle.

#### Halobacteria

##### Phylogeny

Thirteen draft genomes from members of all three known orders within the class *Halobacteria* (as recently defined in Gupta et al., 2014) were obtained from the datasets B1-Br10, Tc-Br11 and PL-Br10 of the most saline soda brines (Table 3). Within the family *Natrialbaceae*, the two drafts B1-Br10_E2g2&27 were affiliated with the genera *Natronoccoccus, Natrialba*, and *Natronobacterium* (Figure 2B; Supplementary Table 5). Since, 16S rRNA sequences belonging to these genera were found in the corresponding amplicon datasets of B1, but their ANI and the ANIs with the three complete reference genomes shown in our genome tree were only between 74.3 and 77.4%, it is not clear whether the drafts belong to one of these three genera or represent one or two novel, distinct genera. Five more drafts of *Natrialbaceae*-related organisms were reconstructed that, based on our genome tree, appeared to be more related to each other than to any other complete genome sequenced from this family. The drafts Tc-Br11_E2g28 and PL-Br10_E2g26 belonged to the same species (ANI = 98.6%) thriving in different brines. Within the family *Haloferacaceae*, the two drafts Tc-Br11_E2g18 and PL-Br10_E2g5 were affiliated with the genus *Halorubrum* (Figure 2C; Supplementary Table 6). Since the latter had an ANI of 80.6% with *Halorubrum lacusprofundi* ATCC 49239, it almost certainly belongs to the genus *Halorubrum*. The three drafts PL-Br10_E2g29&46 and B1-Br10_E2g22 were affiliated with the genus *Halonotius*, but most likely belonged to three distinct, novel genera (ANIs between 69.9 and 74.6%). One draft genome obtained from the trona crystallizer Tc, Tc-Br11_E2g27 belonged to the family *Halobacteriaceae* (Figure 2D; Supplementary Table 7) and comprised a distinct genus related to *Halorhabdus* (ANI = 68.7%).

##### Rhodopsins and metabolic versatility

All reconstructed draft genomes encoded for versatile metabolisms indicating aerobic heterotrophy. Almost all draft genomes possessed a halorhodopsin and/or light-activated signal transducers (sensory rhodopsin I and II) typical of extremely halophilic *Archaea* (Supplementary Table 9). Most drafts encoded a near-complete Krebs cycle and the potential conversion of pyruvate to acetyl CoA by a pyruvate synthase or in some cases by a pyruvate dehydrogenase complex. All drafts encoded subunits I and/or III of a cytochrome *c* oxidase most likely functioning as a terminal oxidase for aerobic respiration (Supplementary Table 10). Almost all genomes encoded a putative L-lactate dehydrogenase (EC 1.1.1.27), which might point to the ability for sugar fermentation. Notably, all of the *Natrialbaceae*-related drafts as well as three of the *Haloferacaceae*-related drafts encoded a tungsten-containing aldehyde:ferredoxin oxidoreductase (AOR; EC 1.2.7.5), a widespread enzyme amongst facultative fermenters (Hagedoorn et al., 2005). Five of the halobacterial drafts encoded the catalytic subunit PsrA of a putative molybdenum-containing thiosulfate/polysulfide reductase (PsrABC; Supplementary Table 10), which can catalyze the quinone-coupled reduction of polysulfides (S${}_{\text{n}}^{2-}$) in the bacterium *Thermus thermophilus* (class *Deinococci*) thriving in geothermal hot springs (Jormakka et al., 2008). Furthermore, membrane-bound polysulfide reductases were expressed during growth of *Halanaeroarchaeum sulfurireducens* HSR2, a sulfidogenic, extreme halophilic, neutrophilic, obligate anaerobic member of the *Halobacteriaceae* capable of dissimilatory reduction of elemental sulfur using acetate as the sole electron donor (Sorokin et al., 2015c). Polysulfides are unstable intermediates formed by the chemical reaction of elemental sulfur with free sulfide in the pH-neutral environment from which *H. sulfurireducens* was obtained. In contrast, thiosulfate and polysulfides are the expected abundantly present sulfur intermediates in alkaline environments such as soda brines. Four of the five PsrABC-containing drafts also encoded rhodanese-like sulfurtransferases (EC:2.8.1.1), which are suggested to be involved in the binding, stabilization and transfer of polysulfides to polysulfide reductase in *H. sulfurireducens* HSR2 (Sorokin et al., 2015c). Additionally, *Haloferacaceae* archaeon Tc-Br11_E2g18 encoded the electron-transfer subunit (NarH) of a putative membrane-bound nitrate reductase (Nar). The molybdoenzyme Nar has been previously shown to function as a terminal electron acceptor in *Haloarcula marismortui* and *Haloferax* sp. (families *Halobacteriaceae* and *Haloferacaceae* respectively) under denitrifying conditions (reviewed by Cabello et al., 2004).

##### Central carbon metabolism

The glycolytic pathway within their central carbon metabolisms appeared to be rather versatile as well. From all three families draft genomes were obtained that encoded a 2-keto-3-deoxygluconate kinase (KDG kinase; EC 2.7.1.45), the key-enzyme of non- or semi-phosphorylative Entner-Doudoroff pathway for glucose degradation (Siebers and Schönheit, 2005), although identifiable glucose dehydrogenases and gluconate dehydratases (enzymes that lead to the formation of 2-keto-3-deoxygluconate) were lacking in our incomplete drafts. Interestingly, most drafts contained a putative glucose/sorbosone dehydrogenase (COG2133), but this enzyme has to our knowledge not been characterized in a member of the *Halobacteria*. Three of the *Natrialbaceae*-related drafts contained within the *Natrialbaceae* encoded a putative non-phosphorylative glyceraldehyde 3-phosphate dehydrogenase (gapN; EC 1.2.1.9), the other ten drafts contained one or both types of phosphorylative glyceraldehyde 3-phosphate dehydrogenase(s), namely GAPDH (EC 1.2.1.12) and/or gap2 (EC 1.2.1.59). The latter enzyme is part of the Emden-Meyerhoff pathway for hexose degradation, which has been shown in *Halococcus saccharolyticus* (family *Halobacteriaceae*) to be the main pathway for fructose degradation (Johnsen et al., 2001). The presence of a similarly branched Entner-Doudoroff/Emden-Meyerhof pathway is suspected to be the general case, since all drafts contained at least one of the enzymes typical for a modified Emden-Meyerhof pathway, which includes an ATP-dependent glucokinase, an ADP-dependent glucokinase/phosphofructokinase and a fructose-bisphosphate aldolase (both Class I and II found).

##### Primary organic carbon degradation

Polysaccharide-degrading potential was found in almost all *Halobacteria*-related draft genomes (Supplementary Table 11). Almost all drafts encoded glucoamylases and related glycoside hydrolases. Putative chitinases were encoded by *Natrialbaceae* archaeon B1-Br10_E2g27 and *Haloferacaceae* archaeon Tc-Br11_E2g18, with catalytic domains resembling those of GH18 and GH19 CAZy family proteins respectively. Putative cellulases were encoded by the *Natrialbaceae* archaea B1-Br10_E2g27, Tc-Br11_E2g14 and Tc-Br11_E2g28, and *Haloferacaceae* archaeon PL-Br10_E2g29. These particular organisms might be able to hydrolyze or even grow on recalcitrant organic substrates such as cellulose and chitin as shown previously for several *Halobacteria* isolated from soda lakes (Sorokin et al., 2015d). Some of these isolates remarkably grew both on celluloses and chitin. Interestingly, also draft genome B1-Br10_E2g27 held both cellulolytic and chitinolytic potential.

##### Aerobic CO oxidation

The draft genome of *Natrialbaceae archaeon* Tc-Br11_E2g1 contained *coxM* and *coxS* genes, the draft of *Natrialbaceae archaeon* Tc-Br11_E2g28 contained a *coxL*. Together these genes encode the three main subunits of a putative aerobic carbon monoxide dehydrogenase (CODH). The large subunit (coxL) of CODH is a molybdopterin dehydrogenase FAD-binding protein with a Mo-Cu sulfur-bridged cluster in its active site that catalyzes the oxidation of CO to CO_2_, yielding two electrons and two protons (Dobbek et al., 2001; Gnida et al., 2003; King and Weber, 2007). While the use of CO as a sole energy and carbon source or as an alternate energy source coupled to CO_2_ fixation (“carboxydotrophy”) is typically restricted to few bacterial groups and can occur only under elevated CO concentrations, the use of CO at low concentrations as an alternative energy source (“carboxydovory”) is suggested to be widespread amongst heterotrophs (King and Weber, 2007; Cunliffe, 2011; King, 2013). It was recently proposed that aerobic CO oxidation might have evolved in the phylum *Euryarchaeota* (genus *Natronorubrum*; King, 2013) and CO consumption has been demonstrated in isolates from the genus *Halorubrum* and *Natronorubrum* (King, 2015). Since, gammaproteobacterial carboxydotrophs as well as carboxydovores have been isolated from several soda lakes around the world (Sorokin et al., 2015a), CO must be to some extent available to soda lake aerobic microbes. As suggested previously for heterotrophic bacteria in the oceans (Martin-Cuadrado et al., 2009), *Halobacteria* might energetically benefit as well from metabolizing CO released by the photolysis of organic material in the surface waters of soda lakes. However, whether any members of the *Euryarchaeota*, including members of the *Halobacteria*, can actually use CO as an energy source remains to be proven.

#### Bacteroidetes

##### Phylogeny

Three Bacteroidetes-related draft genomes were obtained from Tc-Br11 and PL-Br10 (Table 3; Figure 2E; Supplementary Table 8). The draft genome T5-Br10_B2g13 was affiliated with the genera *Gracilimonas* and *Balneola* (order Sphingobacteriales; Chitinophagaceae, no rank; Urios et al., 2006; Choi et al., 2009), is only distantly related to the orders *Flavobacteriales* and *Bacteroidetes Order II Incertae sedis* and had an average GC content of 41%. Similar phylogeny and GC content was reported for *Gracilimonas* and the related genus *Balneola* and annotated genes were affiliated consistently with these two genera (best-hit analysis). While *Gracilimonas*-related OTUs comprised up to 18.84% of total 16S rRNA amplicon sequences from T5, 2.50% of *Chitinophagaceae*-related OTUs could not be assigned to a known genus (Table 2). Since, the ANIs of T5-Br10_B2g13 with the incomplete genomes available from *Balneola vulgaris* DSM 17893 and *Gracilimonas tropica* DSM 19535 were low (65.2 and 66.2% respectively), T5-Br10_B2g13 must belong to a novel genus within the *Chitinophagaceae*. Its closest cultured relative appears to be a proteolytic satellite (bacterial symbiont) of a soda lake filamentous cyanobacterium isolated from the same area (DY Sorokin, unpublished data). Notably in our genome tree, *Gracilimonas, Balneola*, and T5-Br10_B2g13 formed a separate clade from the other three genera within the family *Chitinophagaceae* (namely *Chitinophaga, Niabella*, and *Niastella*).

The draft genome T5-Br10_B2g0 was affiliated with the genus *Psychroflexus* (family *Flavobacteriaceae*; Bowman et al., 1998; Donachie et al., 2004; Yoon et al., 2009) in our genome tree. Since no *Psychroflexus*-related OTUs were found in the *Flavobacteriaceae*-related 16S rRNA amplicon sequences of T5 (Table 2) and ANIs of T5-Br10_B2g0 with the incomplete genomes available from *Psychroflexus gondwanensis* ACAM 44 and *Psychroflexus tropicus* DSM 15496 were low (68.4 and 68.2% respectively), T5-Br10_B2g0 must represent the draft genome of a novel lineage within the *Flavobacteriaceae*.

The draft genome Tc-Br11-B2-g6_7 belonged to the family *Rhodothermaceae* (*Bacteroidetes Order II Incertae sedis*, currently order *Cytophagales*, class *Cytophagia*; Park et al., 2014) branching in a genome tree between the genera *Rhodothermus* and *Salisaeta* of which halophilic isolates have been obtained from marine hot springs and high salt environments respectively (Antón et al., 2002; Vaisman and Oren, 2009). *Rhodothermaceae*-related OTUs without genus assignment as well as *Salisaeta*-related OTUs were found in the 16S rRNA amplicon sequences from Tc (0.24 and 0.06% of total OTUs respectively, Table 2). Since the ANI of Tc-Br-Bacteroidetes-g6_7 with the incomplete genome available from *Salisaeta longa* DSM 21114 was low (67.4%), Tc-Br11-B2-g6_7 belongs to a novel extreme halophilic *Bacteroidetes* lineage within the *Rhodothermaceae*.

##### Central carbon metabolism and primary organic carbon degradation

In all *Bacteroidetes* draft genomes cytochrome c oxidase subunits I and III, marker genes for aerobic respiration, were encoded (Supplementary Table 12), as well as a complete or a near-complete Embden-Meyerhof pathway, pyruvate dehydrogenase complex, and Krebs cycle. Genes encoding both subunits of the 2-oxoglutarate:ferredoxin oxidoreductase (EC 1.2.7.3) as well as a gene encoding a L-lactate dehydrogenase were found for *Chitinophagaceae* bacterium T5-Br10_B2g13 and *Rhodothermaceae* bacterium Tc-Br11_B2g6_7, suggesting they have the potential for sugar fermentation under anoxic or micro-oxic conditions.

Several putative glycoside hydrolases involved in polysaccharide degradation were encoded in the three *Bacteroidetes* draft genomes (Supplementary Table 13). Putative glucoamylases, cellulases, and related enzymes suggest amylolytic and cellulolytic potential for all three organisms. Hemicellulases and related enzymes were only found in *Chitinophagaceae* bacterium T5-Br10_B2g13 and *Rhodothermaceae* bacterium Tc-Br11_B2g6_7. Both *Chitinophagaceae* bacterium T5-Br10_B2g13 and *Flavobacteriaceae* bacterium T5-Br10_B2g0 possessed a putative β-galactosidase (lacZ) and functionally related glycoside hydrolases, as well as chitinase related enzymes (CAZy family GH 23).

##### Denitrification: N_2_O reduction

The genome of *Chitinophagaceae* bacterium T5-Br10_B2g13 encodes a key functional gene *nosZ* for dissimilatory nitrous oxide (N_2_O) reduction within the denitrification pathway (Wood et al., 2001; Jones et al., 2013; Supplementary Table 12). According to the model of *Geobacillus thermodenitrificans*, the gene also lacked a characteristic Tat-signal at the N-terminus for translocation across the membrane (Liu et al., 2008). This finding agrees with the known widespread N_2_O reduction potential in various environments by members of the *Firmicutes* and *Bacteroidetes* which is encoded by a *nosZ* with a presumed Sec-translocation pathway (Jones et al., 2013). Although it is known that some halophilic *Halobacteria* have the capacity to reduce N_2_O (Jones et al., 2013), the process was previously only shown for soda lake isolates within the *Gammaproteobacteria* belonging to the genera *Halomonas, Thioalkalivibrio*, and *Alkalispirillum* (Sorokin et al., 2012, 2014). Amongst the >5 kb contigs we found *nosZ* genes in the three most saline datasets related to the genera *Halorubrum* and *Natronococcus*, but we did not find gammaproteobacterial *nosZ* genes in the >5 kb contigs of T5.

##### Osmotic adaptation: rhodopsins, salt-in strategy and organic osmolytes

Finally, both *Chitinophagaceae* bacterium T5-Br10_B2g13 and *Flavobacteriaceae* bacterium T5-Br10_B2g0 contained a putative sodium pumping rhodopsin with characteristic NQ motif, which could aid in keeping the intracellular sodium ion concentration low (Inoue et al., 2013; Kwon et al., 2013; Supplementary Table 9). *Flavobacteriaceae* bacterium T5-Br10_B2g0 possesses a putative proton pumping proteorhodopsin as well (Béja et al., 2001). Maintaining osmotic balance with high levels of cytoplasmic KCl, often referred to as a “salt-in strategy” (Oren, 2013), appears to be a common strategy for members of the *Halobacteria* and *Nanohaloarchaea* thriving in hypersaline habitats (Ghai et al., 2011; Youssef et al., 2014; Figure 3; Supplementary Figure 3). *Chitinophagaceae* bacterium T5-Br10_B2g13 and *Rhodothermaceae* bacterium Tc-Br11_B2g6_7 seem to deploy this strategy to some extent since their predicted proteomes were enriched in acidic residues compare to that of *E. coli*, despite the fact that putative genes for the synthesis and uptake of organic osmolytes (including betaine aldehyde dehydrogenases and betaine/carnitine/choline transporters) were found in their draft genomes. So far, the “salt-in strategy” was shown only for two bacterial phyla, in the orders *Halanaerobiales* and *Natranaerobiales* (*Firmicutes;* Oren, 2013) and *Salinibacter* (*Bacteroidetes*; Mongodin et al., 2005). No acid-shifted proteome compared to *E. coli* was found for *Flavobacteriaceae* bacterium T5-Br10_B2g0.

#### Natural abundance of the reconstructed genomes

**Figure 4:**
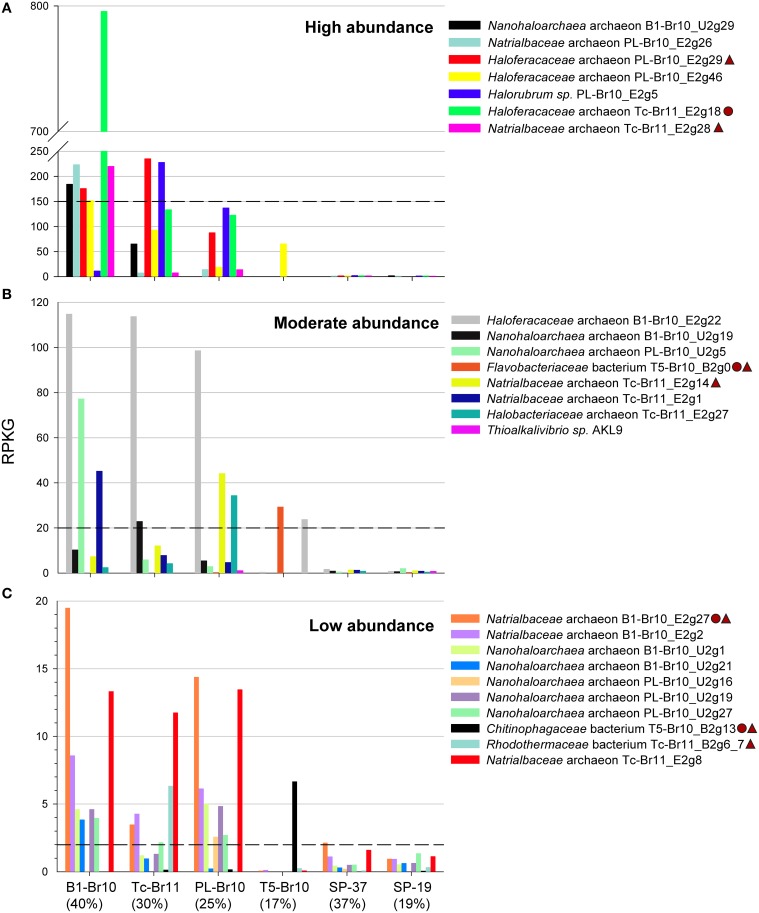
**Read recruitments (< 95% N-identity) of reconstructed draft genomes and a reference draft genome from the genus *Thioalkalivibrio* from different metagenomic datasets**. SP-37 and SP-19 are metagenomes from a crystallizer and an intermediate saline pond of the solar saltern in Santa Pola, Spain, with 37 and 19%, salinity respectively (Ghai et al., 2011). The salinity (in %) for the different soda brine metagenomes is given between brackets. Reconstructed draft genomes encoding putative chitinases are marked in the figure legend with a circle, those encoding putative cellulases with a triangle. RPKG, Reads Per Kilobase of sequenced reads per Gigabase of mapped reads. **(A)** Putative organisms with relatively high abundance (> 150 RPKG), **(B)** with moderate abundance, **(C)** and with relatively low abundance (< 20 RPKG).

In agreement with the 16S amplicon sequencing, *Haloferacaceae*-related genomes were the most abundant in the three most saline brines of B1, Tc and PL (Figure 4). Although the putative chitinolytic *Haloferacaceae archaeon* Tc-Br11_E2g18 was by far the most abundant organism in the brine of B1, the most saline brine still harbored a considerable diverse population of other *Halobacteria* (Figure 4A). The dominant high-GC *Halobacteria* in the most saline brines left their signature in the GC profiles of the raw metagenomic reads (Supplementary Figure 2). *Nanohaloarchaea*-related genomes were confined to the most saline brines and were moderately (reads per kilobase of sequenced reads per gigabase of mapped reads (RPKG) between 120 and 20, Figure 4B) or low abundant (RPKG between 20 and 2, Figure 4C). Previous nanohaloarchaeal metagenomic genome reconstructions were only obtained from more moderate salinities below 250 g/L of total salts (Ghai et al., 2011; Narasingarao et al., 2012; Martínez-García et al., 2014). Only one of the *Halobacteria*-related genomes, *Haloferacaceae* archaeon PL-Br10_E2g46, was moderately abundant in the brine of T5 with a salinity of 170 g/L. It was even more abundant than the *Bacteroidetes*-related genomes that were confined to the brine of T5 (using an arbitrary cut-off of RPKG = 2 for presence of a certain organism within a dataset) and *Thioalkalivibrio* sp. AKL9 (class *Gammaproteobacteria*). *Rhodothermaceae* bacterium Tc-Br11_B2g6_7 had only a low abundance in the brine of Tc. The draft genomes that were reconstructed from a different brine, but were identified to be the same species, *Natrialbaceae* archaeon PL-Br10_E2g26 & Tc-Br11_E2g28 and *Nanohaloarchaea* archaeon B1-Br10_U2g1 and PL-Br10_U2g19, showed comparable RPKG for all four datasets. Finally, only very low read abundance of our soda lake draft genomes was found in the metagenomics datasets SP-37 and SP-19, originating from salterns with neutral pH. Although *Halobacteria, Nanohaloarchaea*, and *Bacteroidetes* are common taxa in high-salt environments, the specific organisms belonging to these groups that can thrive at high alkalinities are likely to differ from those that thrive at circum-neutral pH. The same holds true for pure cultures, where the isolates from soda lakes in most cases differentiate from their halophilic counterparts on the genus level (Sorokin et al., 2014, 2015a).

## Conclusions and future research

Salinity has a similar dominant influence on the overall prokaryote community structure of alkaline soda lake brines as it has on that of hypersaline ponds of solar salterns with neutral pH; over a salt concentration of roughly 250 g/L bacterial abundance declines and dominance shifts to members of the *Halobacteria* (Rodriguez-Valera et al., 1985; Oren, 1994; Ghai et al., 2011). Yet hypersaline soda lake brines with salinities exceeding 250 g/L harbor considerably different and more diverse communities compared to solar saltern brines of similar salinities. The absence of calcium salts that precipitate at high pH and much lower prevalent magnesium concentrations could offer explanations for the differences found in the community structure. Furthermore, the investigated, shallow soda lakes from the Kulunda Steppe have a very unstable water regime with large fluctuations in salinity throughout the seasons and the year, according to local snow melt, groundwater inflow and evaporation rates. These fluctuations could also contribute to sustaining such diverse soda brine communities. Finally, the nearly 50% lower osmotic pressure in hypersaline soda lake brines compared to solar salterns (Banciu and Sorokin, 2013; Sorokin et al., 2015a) might be the reason why in particular *Nanohaloarchaea*, which rely solely on the salt-in strategy for osmotic adaption, prefer higher salinities in alkaline brines than in NaCl brines with circum-neutral pH.

By reconstructing the first draft genomes from members of the candidate class *Nanohaloarchaea* present in hypersaline soda brines, we extend the amount of available genomic data from this enigmatic new group of *Archaea* considerably and suggest that their inferred ecological lifestyle with respect to oxygen respiration should be revised. Furthermore, we identified hydrolytic capacity not only within previously unrecognized members of the *Bacteroidetes*, but also amongst members of the *Halobacteria* which outnumber the first in the most saline brines. The fact that some members of the *Halobacteria* might fulfill a primary rather than a secondary degrading function in the organic carbon cycle of soda lakes is supported by previous culture-dependent studies. Overall, the dominant organisms in the most saline brines seem to be aerobic heterotrophs that adapted to the presence of light and to the low solubility of oxygen at high salinities with putative sugar fermentative capabilities. We identified two members of the *Bacteroidetes* that might utilize light not simply as an additional energy source or sensory input for movement, but rather to pump out Na^+^ via sodium pumping rhodopsins.

The presented genomic data provide a first insight into the physiological potential of a wealth of still uncultured organisms living in hypersaline soda lake brines. We hope this study can pave the way for the design of novel isolation strategies and protein structure-function studies, crucial in obtaining unequivocal evidence for the proposed metabolic features of the novel organisms. More in depth sampling over time or space of soda brines could bring a more detailed understanding of the influence of environmental gradients in salinity and salt composition, as well as of other parameters such as pH and seasonality, on specific microbial populations and functional groups. Future research should combine transcriptomic and/or proteomic approaches with *in situ* rates of element transformations to reveal the active prokaryote community members in hypersaline soda lake brines in relation to salinity and could aim at verifying the functional role and relative importance of rare vs. abundant community members.

## Author contributions

DS, PH, and GM were responsible for the conception of the work. DS collected the samples and extracted the DNA. GM prepared samples for amplicon sequencing and performed subsequent analysis. ST was responsible for the DNA sequencing. RG performed the assembly of the metagenomic reads and assisted CV with the genome reconstructions and subsequent analysis. CV integrated and interpreted all data and is the primary author of the manuscript. She received thereby substantial contributions of all co-authors, including final approval for this version to be published.

## Funding

CV and GM are supported by the ERC Advanced Grant PARASOL (No. 322551). The work of CV was further supported by an STSM Grant from the COST Action ES1103. RG is partially supported by the Grant Agency of the Czech Science Foundation under the research grant 13-00243S. FR-V and RG are supported by a grant from the European Union (MaCuMBA project, grant 311975). DS is supported by the Russian Foundation for Basic Research (16-04-00035). The work conducted by the U.S. Department of Energy Joint Genome Institute, a DOE Office of Science User Facility, is supported under Contract No. DE-AC02-05CH11231.

### Conflict of interest statement

The authors declare that the research was conducted in the absence of any commercial or financial relationships that could be construed as a potential conflict of interest.
