# Supplementary material for: Metagenomic Insights into the Uncultured Diversity and Physiology of Microbes in Four Hypersaline Soda Lake Brines
Source: Front Microbiol. 2016 Feb 25;7:211. doi: 10.3389/fmicb.2016.00211 (PMC4766312; doi:10.3389/fmicb.2016.00211)
Supplement: Supplementary file 1 [file DataSheet1.pdf]

Supplementary Material

Metagenomic insights into the uncultured diversity and physiology of microbes in four hypersaline soda lake brines

Charlotte D. Vavourakis, Rohit Ghai, Francisco Rodriguez-Valera, Dimitry Y. Sorokin, Susannah G. Tringe, Philip Hugenholtz, Gerard Muyzer*

*** Correspondence:** Gerard Muyzer: g.muijzer@uva.nl

1 Supplementary Figures

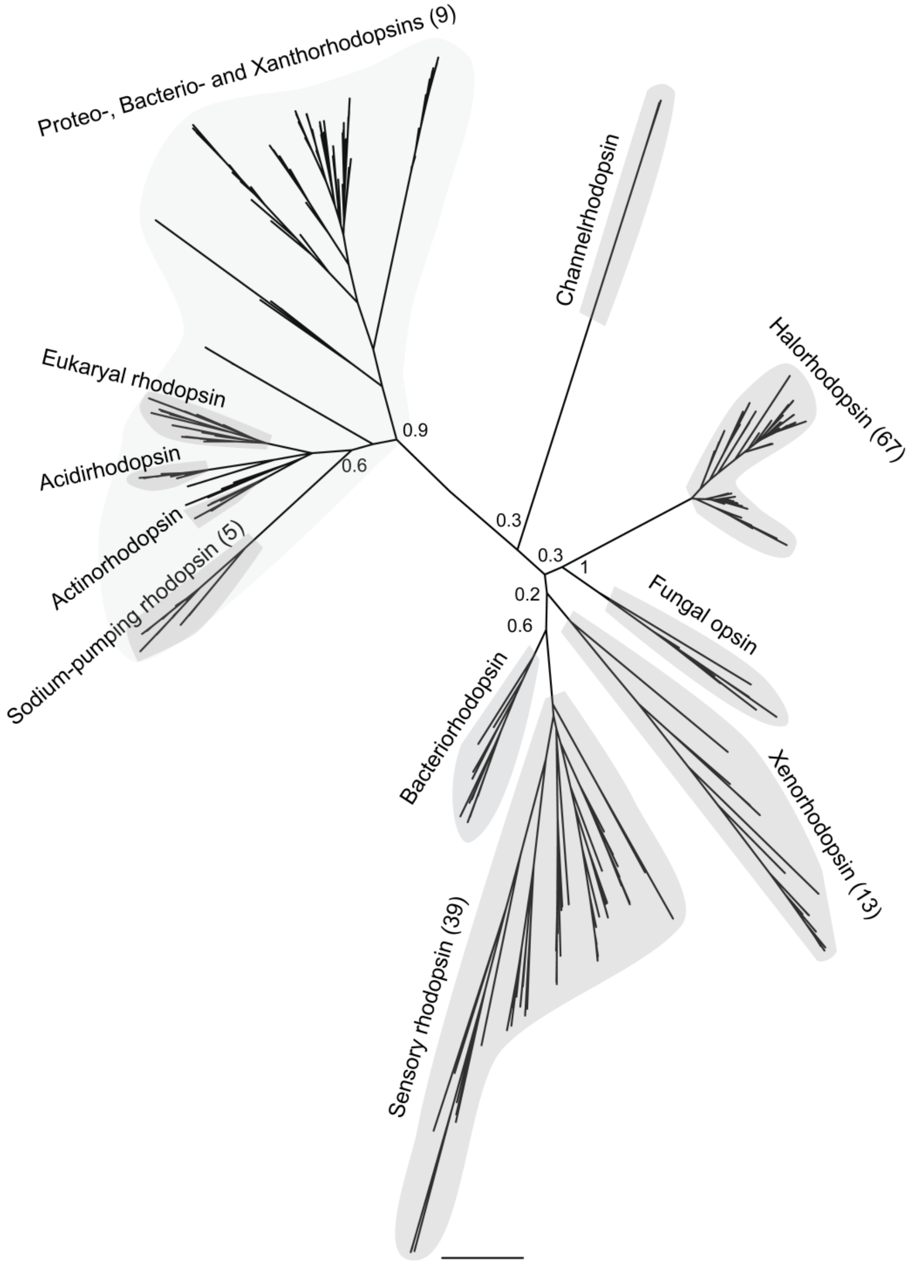

Supplementary Figure 1. Maximum-likelihood tree of rhodopsins found on the >5kb contigs from the soda brine datasets and reference protein sequences. The number of rhodopsins found in the soda brine datasets within a certain functional group is given between brackets. Putative rhodopsins from viral reference genomes were pruned from the tree. Scale bar indicates 9% sequence difference. Selected bootstraps values (100x) are shown at the nodes.

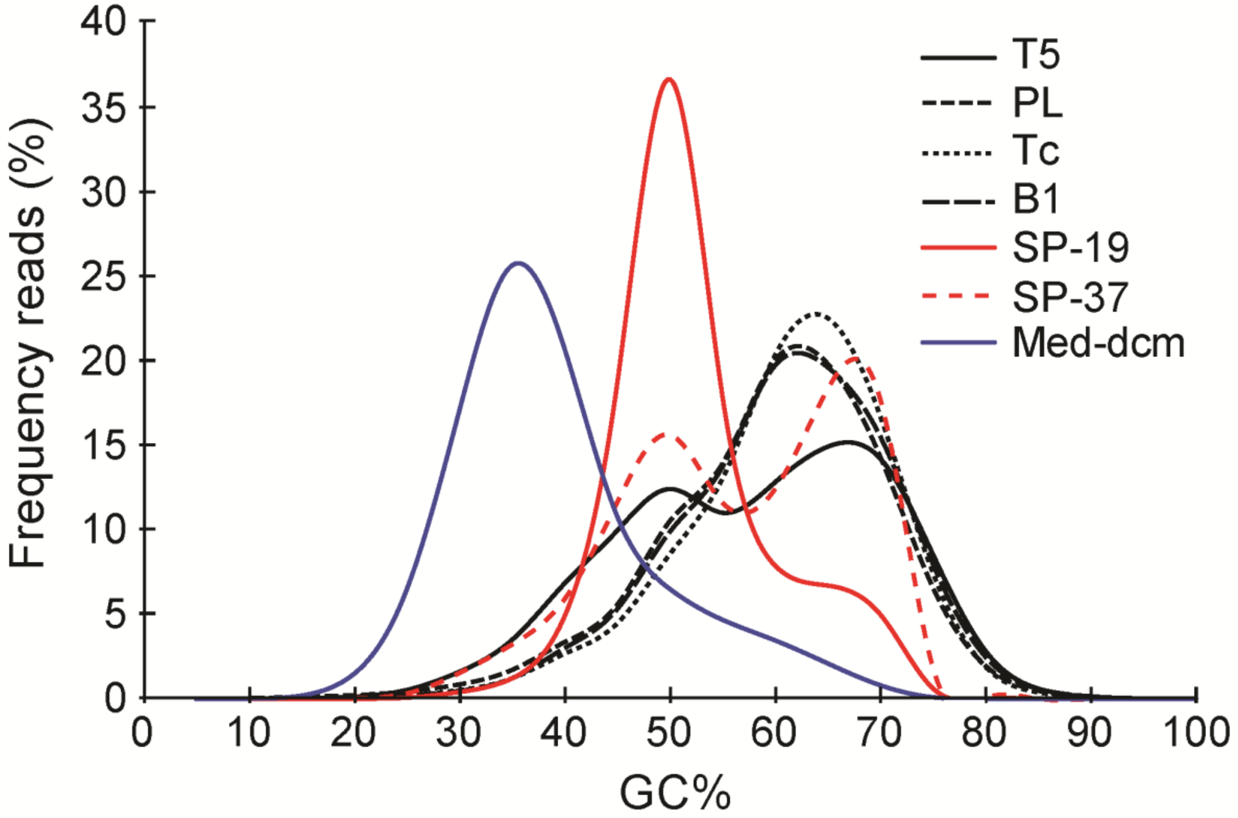

Supplementary Figure 2. Relative frequency of GC% of a subsample of 1 million sequence reads from the Kulunda soda brines (Tanatar-5 (T5, Salinity S=17%), Picturesque Lake (PL, S=25%), Tanatar trona crystallizer (Tc, S=30%) and Bitter-1 (B1, S=40%) calculated in intervals of bin width 5 and compared to the GC% distribution of three metagenomes obtained by 454 pyrosequencing from saline environments with neutral pH; a crystallizer pond (SP-37, S=37%) and an intermediate saline pond (SP-19, S=19%) of the solar saltern in Santa Pola, Spain (Ghai *et al*., 2011) and the deep chlorophyll maximum in the Mediterranean Sea near Alicante, Spain (Med-dcm, 3.8% salinity; Ghai *et al*., 2010). The large peak in the GC-profile of SP-37 was attributed to the predominance of the square archaeon *Haloquadratum walsbyi* that has a low average GC-content compared to other members of the *Halobacteria,* such as those that dominate in the hypersaline soda brines.

Additional reference:

Ghai, R., Martin-Cuadrado, A., Molto, A.G., Heredia, I.G., Cabrera, R., Martin, J., *et al*. (2010). Metagenome of the Mediterranean deep chlorophyll maximum studied by direct and fosmid library 454 pyrosequencing. *ISME J.* 4, 1154-1166. doi:10.1038/ismej.2010.44

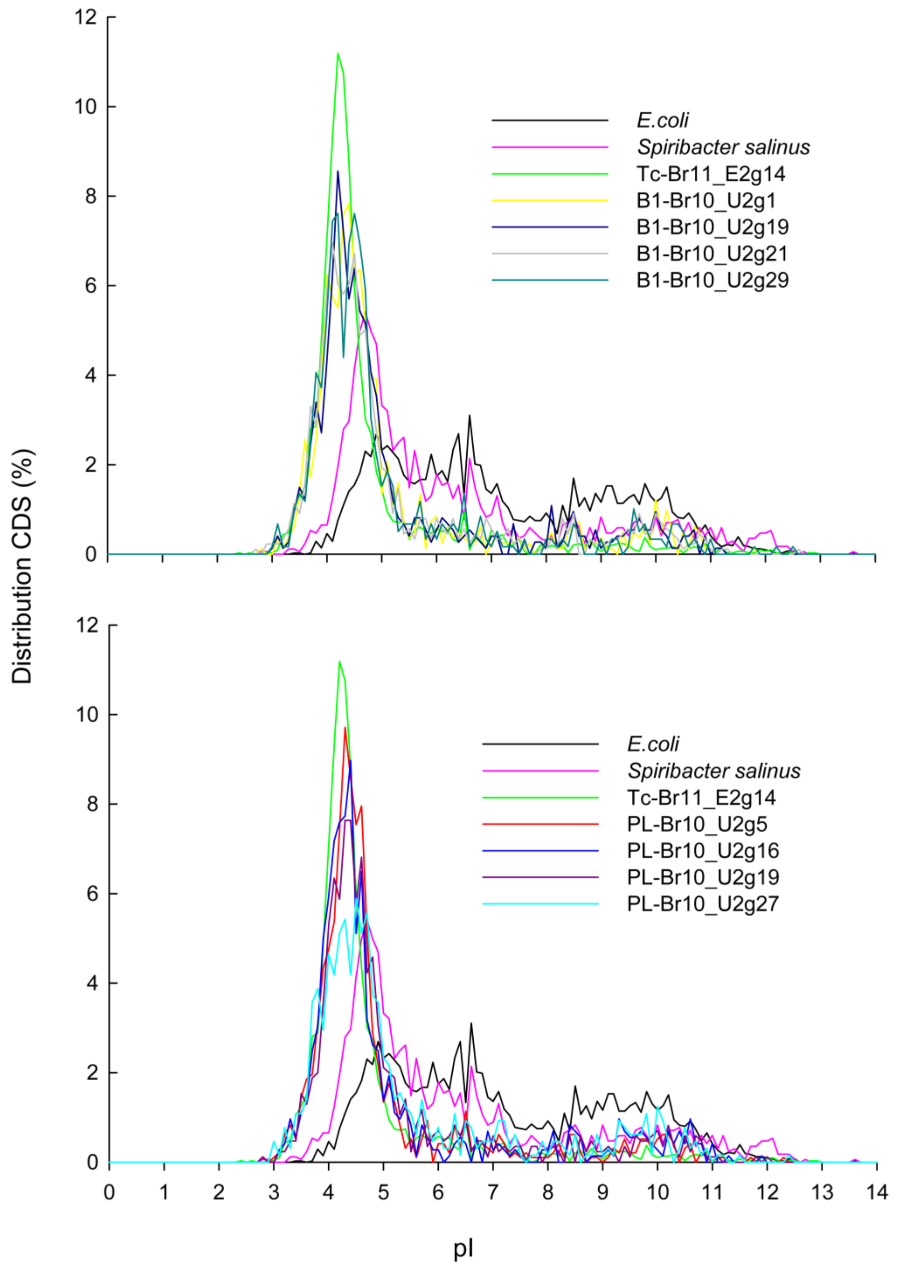

Supplementary Figure 3. Predicted isoelectric points (pI) for the coding sequences (CDS) of the *Nanohaloarchaea* draft genomes demonstrating the effect of the 'salt-in' strategy for osmotic adaptation on their protein composition. The pI profiles of *E.coli* ('salt-out'), Tc-Br11_E2g14 (family *Natrialbaceae*, archaeal 'salt-in') and *Spiribacter salinus* (bacterial 'salt-in'/mixed strategy) are plotted for comparison.

2 Supplementary Tables

Supplementary Table 1. Metagenomics contigs >5kb binned to top level taxa. Metagenomes of the brines of Lake Tanatar 5 (T5-Br10), Picturesque Lake (PL-Br10), Tanatar crystalizer (Tc-Br11) and Lake Bitter 1 (B1-Br10).

	**T5-Br10**	**PL-Br10**	**Tc-Br11**	**B1-Br10**
Acidobacteria	8	3	1	4
Actinobacteria	1304	430	27	176
Alphaproteobacteria	2530	488	40	269
Aquificae	0	1	0	0
Bacteroidetes	546	349	256	11
Betaproteobacteria	113	61	13	36
Caldiserica	0	0	1	0
Chloroflexi	13	10	4	3
Chrysiogenetes	2	0	0	0
Crenarchaeota	2	7	5	5
Cyanobacteria	65	36	13	18
Deferribacteres	2	0	0	1
Deinococcus-Thermus	10	7	1	6
Deltaproteobacteria	118	258	185	23
Dictyoglomi	0	0	0	1
Epsilonproteobacteria	8	2	1	2
Eukaryota	8963	7763	41	6811
Euryarchaeota	222	10152	6442	4931
Firmicutes	211	907	788	389
Fusobacteria	1	2	1	1
Gammaproteobacteria	2246	1335	393	436
Gemmatimonadetes	56	7	2	0
Nanoarchaeota	0	1	0	1
Nitrospirae	1	1	1	1
Planctomycetes	16	5	5	3
Spirochaetes	11	7	2	34
Synergistetes	0	6	1	0
Tenericutes	149	3	1	49
Thaumarchaeota	0	6	4	4
Thermodesulfobacteria	1	0	0	0
Thermotogae	1	3	2	2
Unclassified (incl. *Nanohaloarchaea*)	2028	2685	896	2023
Verrucomicrobia	326	6	3	4
Viruses	203	168	68	101
Discarded	194	389	229	206
Total >5kb contigs	19350	25098	9426	15551

Supplementary Table 2. GenBank Accesion numbers of the selected reconstructed draft genomes discussed in this paper.

Name <Id>	GenBank Accesion
Chitinophagaceae bacterium T5-Br10_B2g13	LKNA00000000
Flavobacteriaceae bacterium T5-Br10_B2g0	LKMZ00000000
Rhodothermaceae bacterium Tc-Br11_B2g6_7	LKNB00000000
Halobacteriaceae archaeon Tc-Br11_E2g27	LKNH00000000
Haloferacaceae archaeon PL-Br10_E2g46	LKMT00000000
Haloferacaceae archaeon B1-Br10_E2g22	LKML00000000
Haloferacaceae archaeon PL-Br10_E2g29	LKMS00000000
Halorubrum sp. PL-Br10_E2g5	LKMU00000000
Haloferacaceae archaeon Tc-Br11_E2g18	LKNE00000000
Nanohaloarchaea archaeon B1-Br10_U2g1	LKMN00000000
Nanohaloarchaea archaeon B1-Br10_U2g21	LKMP00000000
Nanohaloarchaea archaeon B1-Br10_U2g19	LKMO00000000
Nanohaloarchaea archaeon B1-Br10_U2g29	LKMQ00000000
Nanohaloarchaea archaeon PL-Br10_U2g5	LKMY00000000
Nanohaloarchaea archaeon PL-Br10_U2g19	LKMW00000000
Nanohaloarchaea archaeon PL-Br10_U2g16	LKMV00000000
Nanohaloarchaea archaeon PL-Br10_U2g27	LKMX00000000
Natrialbaceae archaeon B1-Br10_E2g2	LKMK00000000
Natrialbaceae archaeon B1-Br10_E2g27	LKMM00000000
Natrialbaceae archaeon Tc-Br11_E2g1	LKNC00000000
Natrialbaceae archaeon Tc-Br11_E2g14	LKND00000000
Natrialbaceae archaeon Tc-Br11_E2g28	LKNF00000000
Natrialbaceae archaeon Tc-Br11_E2g8	LKNG00000000
Natrialbaceae archaeon PL-Br10_E2g26	LKMR00000000

Supplementary Table 3. Total richness of top level taxa and OTUs detected in the metagenomic and amplicon datasets from four different soda lake brines. Lake Bitter 1 (B1; Salinity S = 400 g/L), Tanatar crystalizer (Tc; S = 300g/L), Picturesque Lake (PL; S = 250g/L) and Lake Tanatar 5 (T5; S = 170 g/L). # = total number of.

	B1-Br10	Tc-Br11	PL-Br10	T5-Br10
Metagenomes - # top level taxa	6	9	10	14
Amplicon sequences - # top level taxa	12	13	19	11
Amplicon sequences - # genus level OTUs	47	46	102	34

Supplementary Table 4. Average nucleotide identity (ANI) matrix of the *Nanohaloarchaea*-related draft (1-4, 9-12) and reference (5-8) genomes.

	(1)	(2)	(3)	(4)	(5)	(6)	(7)	(8)	(9)	(10)	(11)	(12)
B1-Br10_U2g1 (1)	100											
B1-Br10_U2g19 (2)	74.2	100										
B1-Br10_U2g21 (3)	66.8	67.2	100									
B1-Br10_U2g29 (4)	67.0	67.1	69.0	100								
Candidatus Haloredivivus sp G17' (5)	66.9	67.4	68.6	71.9	100							
Candidatus Nanosalina sp J07AB43' (6)	66.3	66.3	65.9	66.8	66.7	100						
Candidatus Nanosalinarum sp J07AB56' (7)	63.9	64.1	62.8	63.3	64.3	64.6	100					
Nanohaloarchaea archaeon AB578-D14 (8)	63.3	63.6	61.6	63.1	65.0	64.6	81.1	100				
PL-Br10_U2g16 (9)	67.8	68.0	66.0	66.6	67.3	66.2	64.0	62.4	100			
PL-Br10_U2g19 (10)	99.4	74.5	66.7	66.8	67.1	66.3	64.2	63.4	68.0	100		
PL-Br10_U2g27 (11)	67.0	67.6	74.8	69.2	70.1	66.1	64.0	63.4	66.3	67.1	100	
PL-Br10_U2g5 (12)	66.8	67.3	68.7	74.9	73.5	66.3	63.5	62.2	66.2	66.7	69.2	100

Supplementary Table 5. ANI matrix of the *Natrialbaceae*-related draft (1-2, 7-11) and reference (3-6) genomes.

	(1)	(2)	(3)	(4)	(5)	(6)	(7)	(8)	(9)	(10)	(11)
B1-Br10_E2g2 (1)	100										
B1-Br10_E2g27 (2)	76.5	100									
Halovivax ruber XH 70 (3)	74.1	71.4	100								
Natrialba magadii (4)	77.3	75.1	73.9	100							
Natronobacterium gregoryi SP2 (5)	77.2	74.3	73.7	77.4	100						
Natronococcus occultus SP4 (6)	77.5	74.4	74.1	77.3	77.1	100					
PL-Br10_E2g26 (7)	73.7	72.5	72.4	73.9	73.2	73.3	100				
Tc-Br11_E2g1 (8)	76.3	73.8	73.8	75.6	75.2	75.9	73.6	100			
Tc-Br11_E2g14 (9)	74.7	72.9	73.2	74.5	74.1	74.7	76.2	74.4	100		
Tc-Br11_E2g28 (10)	73.5	72.2	72.1	73.7	73.1	72.9	98.6	73.5	76.1	100	
Tc-Br11_E2g8 (11)	76.0	73.1	73.4	74.6	74.6	75.5	73.6	76.1	74.5	73.1	100

Supplementary Table 6. ANI matrix of the *Haloferacaceae*-related draft (1, 4-7) and reference (2-3) genomes.

	(1)	(2)	(3)	(4)	(5)	(6)	(7)
B1-Br10_E2g22 (1)	100						
Halonotius pteroides (2)	72.7	100					
Halorubrum lacusprofundi ATCC 49239 (3)	70.0	72.0	100				
PL-Br10_E2g29 (4)	74.8	74.6	71.6	100			
PL-Br10_E2g46 (5)	68.7	68.8	68.3	69.9	100		
PL-Br10_E2g5 (6)	69.5	71.2	80.6	71.6	68.4	100	
Tc-Br11_E2g18 (7)	69.4	70.9	76.9	71.6	68.7	76.4	100

Supplementary Table 7. ANI matrix of the *Halobacteriaceae*-related draft (4) and reference (1-3) genomes.

	(1)	(2)	(3)	(4)
Halomicrobium mukohataei DSM12286 (1)	100			
Halorhabdus tiamatea SARL4B (2)	73.0	100		
Halorhabdus utahensis DSM 12940 (3)	72.4	83.9	100	
Tc-Br11_E2g27 (4)	68.7	68.1	68.0	100

Supplementary Table 8. ANI matrix of the *Bacteroidetes*-related draft (11-13, 1-10) and reference (3-6) genomes.

	(1)	(2)	(3)	(4)	(5)	(6)	(7)	(8)	(9)	(10)	(11)	(12)	(13)
Balneola vulgaris DSM 17893 (1)	100												
Gracilimonas tropica DSM 19535 (2)	67.3	100											
Gramella_forsetii KT0803 (3)	63.2	62.8	100										
Psychroflexus gondwanensis ACAM 44 (4)	62.9	62.4	66.9	100									
Psychroflexus tropicus DSM 15496 (5)	62.4	62.0	67.0	73.6	100								
Rhodothermus marinus DSM 4252 (6)	61.7	62.0	61.0	61.0	59.9	100							
Rhodothermus marinus SG0.5JP17 172 (7)	61.8	61.9	60.8	60.9	60.0	95.0	100						
Salinibacter ruber DSM 13855 (8)	61.6	61.3	61.9	61.7	59.6	67.8	67.8	100					
Salinibacter ruber M8 (9)	61.4	61.5	61.0	60.9	59.1	67.8	67.8	98.5	100				
Salisaeta longa DSM 21114 (10)	62.1	61.7	60.6	61.0	60.0	67.8	67.8	69.9	69.8	100			
T5-Br10_B2g0 (11)	62.4	61.7	67.0	68.4	68.2	59.0	58.9	57.2	56.8	58.0	100		
T5-Br10_B2g13 (12)	65.2	66.2	61.6	61.7	61.5	61.6	61.7	61.2	61.3	61.4	61.3	100	
Tc-Br11_B2g6_7 (13)	61.9	62.3	60.7	60.9	59.5	67.6	67.6	67.1	67.2	67.4	58.8	62.1	100

Supplementary Table 9. Functional classification of rhodopsin sequences found in the reconstructed draft genomes as derived from the phylogenetic protein tree shown in Supplementary Figure 1

Taxon	Draft ID	Sequence ID	Functional classification
Bacteroidetes	T5-Br10_B2g0	LT5-BRINE-C1367-cds1	sodium-pumping proteorhodopsin
	T5-Br10_B2g0	LT5-BRINE-C2167-cds1	proteorhodopsin
	T5-Br10_B2g13	LT5-BRINE-C29-cds9	sodium-pumping proteorhodopsin
Nanohaloarchaea	B1-Br10_U2g21	LB-BRINE-C50-cds41	xenorhodopsin
	B1-Br10_U2g19	LB-BRINE-C34-cds1	xenorhodopsin
	B1-Br10_U2g29	LB-BRINE-C1247-cds2	xenorhodopsin
	PL-Br10_U2g19	PL-BRINE-C3753-cds9	xenorhodopsin
	PL-Br10_U2g16	PL-BRINE-C117-cds14	xenorhodopsin
	PL-Br10_U2g27	PL-BRINE-C793-cds5	xenorhodopsin
Natrialbaceae	B1-Br10_E2g2	LB-BRINE-C355-cds25	halorhodopsin
	B1-Br10_E2g2	LB-BRINE-C1970-cds5	sensory rhodopsin
	B1-Br10_E2g2	LB-BRINE-C4658-cds5	sensory rhodopsin-2
	B1-Br10_E2g27	LB-BRINE-C5464-cds6	halorhodopsin
	PL-Br10_E2g26	PL-BRINE-C16264-cds6	halorhodopsin
	Tc-Br11_E2g1	LTtc-BRINE-C6008-cds8	halorhodopsin
	Tc-Br11_E2g14	LTtc-BRINE-C69-cds40	halorhodopsin
	Tc-Br11_E2g14	LTtc-BRINE-C6857-cds7	sensory rhodopsin-2
	Tc-Br11_E2g28	LTtc-BRINE-C2288-cds2	halorhodopsin
	Tc-Br11_E2g8	LTtc-BRINE-C596-cds1	halorhodopsin
Haloferacaceae	B1-Br10_E2g22	LB-BRINE-C150-cds31	sensory rhodopsin-2
	PL-Br10_E2g46	PL-BRINE-C5937-cds4	sensory rhodopsin-2
	Tc-Br11_E2g18	LTtc-BRINE-C3325-cds9	sensory rhodopsin-1
	Tc-Br11_E2g18	LTtc-BRINE-C7190-cds3	halorhodopsin
Halobacteriaceae	Tc-Br11_E2g27	LTtc-BRINE-C497-cds20	sensory rhodopsin
	Tc-Br11_E2g27	LTtc-BRINE-C5242-cds3	sensory rhodopsin-2

Supplementary Table 10. Detected marker genes involved in carbon (C), nitrogen (N) and sulfur (S) metabolism from selected *Halobacteria*-related draft genomes from a selection of draft genomes. Marker genes were selected according to Lauro *et al*. (2011) and Llorens-Marès *et al*. (2015). *Haloferacaceae*-, *Halobacteriaceae*- and *Natrialbaceae*-related draft genomes are marked in light grey, light blue and light pink respectively.

	Functional process	Knumber	Marker gene	Tc-Br11_E2g27	B1-Br10_E2g22	PL-Br10_E2g29	PL-Br10_E2g46	PL-Br10_E2g5	Tc-Br11_E2g18	B1-Br10_E2g27	B1-Br10_E2g2	PL-Br10_E2g26	Tc-Br11_E2g14	Tc-Br11_E2g1	Tc-Br11_E2g28	Tc-Br11_E2g8
**C**	Anaerobic carbon fixation	K00174	2-oxoglutarate:ferredoxin oxidorecutase subunit alpha	+	+	+	+	+	+		+	+	+	+	+	+
		K00175	2-oxoglutarate:ferredoxin oxidoreductase subunit beta	+	+	+	+	+	+	+	+	+	+	+	+	+
		K00244	fumarate reductase flavoprotein subunit	+										+		
	Fermentation	K00016	L-lactate dehydrogenase	+	+			+	+	+	+	+	+	+	+	+
	Aerobic respiration	K02274	cytochrome c oxidase subunit I (coxa)	+	+		+		+	+	+	+	+	+	+	+
		K02276	cytochrome c oxidase subunit III (coxc)		+	+						+			+	
	CO oxidation	K03518	CO dehydrogenase small subunit (coxS)											+		
		K03519	CO dehydrogenase medium subunit (coxM)											+		
		K03520	CO dehydrogenase large subunit (coxL)												+	
**N**	Nitrogen assimilation	K01915	glutamine syntetase (glnA)	+	+	+		+	+		+		+	+	+	+
		K00265	glutamate synthase (NADPH/NADH) large chain (gltB)	+	+					+	+	+	+			+
	Mineralization	K00260	glutamate dehydrogenase													+
		K00261	glutamate dehydrogenase								+			+		
		K00262	glutamate dehydrogenase	+	+			+	+	+	+	+	+	+	+	+
	Nitrate reduction + nitrite oxidation	K00371	nitrate reductase beta & nitrite oxidoreductase (narH/nxrB)						+							
**S**	Assimilatory sulfate reduction	K00957	sulfate adenylyltransferase subunit 2 (cysD)						+		+		+		+	+
	Mineralization	K01011	3-mercaptopyruvate sulfurtransferase	+	+		+			+			+	+		+
	Polysulfide reduction	K08352	polysulfide reductase chain A (psrA)		+			+		+			+	+		

Supplementary Table 11. Putative glucoside hydrolases (GHs) identified in the gene repertoire from *Halobacteria*-related draft genomes. Predicted protein sequences were selected by sequence based annotation and Pfam rule based annotation using the CAZymes Analysis Toolkit (CAT; E-value 0.001, Bit-score 55). Unless the Contig-ID is marked with an asterix (*), query and subject sequences had a consistent length and Pfam domain(s). Query Pfam domains and subject GenBank sequence identifiers (GI), as well as putative gene products of the besthits (Blast-N against; all e-values < 1E-05) and COG identifiers are given. CAZy families likely involved in polysaccharide degradation include chitinases (light green), cellulases and related glucanases (yellow), hemicellulases and related enzymes (dark red), glucoamylases and related GHs (orange). GH families likely involved in the hydrolysis of mono- and disaccharides are marked blue. Results classified under CAZy families GH 109 and GH 93 (encoding putative oxidoreductases and transcriptional regulators) respectively are not shown. [1]some enzymes belonging to this family might hydrolyse hemicellulose (e.g. xylanases), mono- or disaccharides. *Haloferacaceae*-, *Halobacteriaceae*- and *Natrialbaceae*-related draft genomes are marked in light grey, light blue and light pink respectively.

Draft	ContigID-cds#	Putative Gene product	COG	Query-Pfam domains	Subject GI	CAZy Family
PL-Br10_E2g46	PL-BRINE-C7746-cds1	alpha amylase		Alpha-amylase	ADE04704.1	GH13
	PL-BRINE-C7746-cds2	glucoamylase related GH		Glucodextran_N|Glyco_hydro_15	AFK19507.1	GH15
B1-Br10_E2g22	LB-BRINE-C1345-cds8	hypothetical protein			ACM56147.1	GH15
	LB-BRINE-C1345-cds9	GH		Alpha-amylase	ADE03517.1	GH13
	LB-BRINE-C1416-cds3	GH family 15	SGA1 COG3387 612 Glucoamylase and related GHs	Glyco_hydro_15	AFZ73398.1	GH15
	LB-BRINE-C48-cds22	4-alpha-glucanotransferase	MalQ COG1640 520 4-alpha-glucanotransferase	Glyco_hydro_77	ADQ67101.1	GH77
	LB-BRINE-C72-cds35	glucoamylase related GH		Glucodextran_N|Glyco_hydro_15	AFK19507.1	GH15
	LB-BRINE-C72-cds36	alpha amylase		Alpha-amylase	ADE04704.1	GH13
	LB-BRINE-C792-cds10	GH	AmyA COG0366 505 GHs	Alpha-amylase	AFK18760.1	GH13
PL-Br10_E2g29	PL-BRINE-C7110-cds5	hypothetical protein		Glyco_hydro_3|Glyco_hydro_3_C|Fn3-like	AGB37354.1	GH3
	PL-BRINE-C11462-cds3	glucoamylase related GH			ABU58666.1	GH15
	PL-BRINE-C2927-cds3	GH	AmyA COG0366 505 GHs	Alpha-amylase	AFK18760.1	GH13
	PL-BRINE-C7633-cds4	4-alpha-glucanotransferase	MalQ COG1640 520 4-alpha-glucanotransferase	Glyco_hydro_77	ADQ67101.1	GH77

Draft	ContigID-cds#	Putative Gene product	COG	Query-Pfam domains	Subject GI	CAZy Family
PL-Br10_E2g5	PL-BRINE-C3624-cds5	GH family 68		Glyco_hydro_68	ACM58061.1	GH68
Tc-Br11_E2g18	LTtc-BRINE-C3859-cds7	hypothetical protein		DUF4157	AFZ68540.1	GH19
	LTtc-BRINE-C1915-cds10	glucan 14-alpha-glucosidase			ACM56147.1	GH15
	LTtc-BRINE-C2405-cds4	4-alpha-glucanotransferase	MalQ COG1640 520 4-alpha-glucanotransferase	Glyco_hydro_77	ACM57093.1	GH77
	LTtc-BRINE-C3355-cds9	Levansucrase		Glyco_hydro_68	ACV48051.1	GH68
B1-Br10_E2g27	LB-BRINE-C948-cds17*	Chitinase		CBM_5_12|Glyco_hydro_18	ADB61056.1	GH18
	LB-BRINE-C859-cds10	hypothetical protein			AGF58675.1	GH51[1]
	LB-BRINE-C5385-cds6	alpha-D-1,4-glucosidase		Alpha-amylase	ACV11235.1	GH13
	LB-BRINE-C7011-cds1	glucoamylase		Glucodextran_N|Glyco_hydro_15	AAV46829.1	GH15
	LB-BRINE-C7011-cds3	4-alpha-glucanotransferase	MalQ COG1640 520 4-alpha-glucanotransferase	Glyco_hydro_77	ACV11232.1	GH77
B1-Br10_E2g2	LB-BRINE-C1245-cds16	GH family 15	SGA1 COG3387 612 Glucoamylase and related GHs	Glyco_hydro_15	AFZ73398.1	GH15
Tc-Br11_E2g1	LTtc-BRINE-C1785-cds15	GH family 2			AFZ71909.1	GH2
Tc-Br11_E2g14	LTtc-BRINE-C909-cds8	hypothetical protein			BAM03099.1	GH74[1]
	LTtc-BRINE-C1440-cds11	glucan 14-alpha-glucosidase			AEM58374.1	GH15
	LTtc-BRINE-C5756-cds2	GH family 15	SGA1 COG3387 612 Glucoamylase and related GHs	Glyco_hydro_15	AGB38313.1	GH15
	LTtc-BRINE-C62-cds4	cyclomaltodextrinase	AmyA COG0366 505 GHs	Alpha-amylase_N|Alpha-amylase	AEH38729.1	GH13|CBM34
	LTtc-BRINE-C9-cds29	GH family 81	ACF2 COG5498 760 Predicted GH	Glyco_hydro_81	AEH38967.1	GH81

Draft	ContigID-cds#	Putative Gene product	COG	Query-Pfam domains	Subject GI	CAZy Family
Tc-Br11_E2g14 (continued)	LTtc-BRINE-C123-cds11	GH			AFZ71909.1	GH2
	LTtc-BRINE-C1530-cds7	Levansucrase		Glyco_hydro_68	AGB39317.1	GH68
	LTtc-BRINE-C44-cds28	GH family 42	LacA COG1874 673 Beta-galactosidase	Glyco_hydro_42|Glyco_hydro_42M	AGB38905.1	GH42
	LTtc-BRINE-C755-cds18	alpha-galactosidase	CelF COG1486 442 Alpha-galactosidases/6-phospho-beta-glucosidases, family 4 of GHs	Glyco_hydro_4|Glyco_hydro_4C	AEH38218.1	GH4
Tc-Br11_E2g28	LTtc-BRINE-C4350-cds3*	GH family 3		Glyco_hydro_3|Glyco_hydro_3_C|Fn3-like	ADQ68885.1	GH3
	LTtc-BRINE-C1834-cds10	alpha-galactosidase		Glyco_hydro_31	ACM58429.1	GH36
	LTtc-BRINE-C653-cds2	glucan 1 4-alpha-glucosidase /GH			AAV47172.1	GH15
	LTtc-BRINE-C653-cds7	cyclomaltodextrinase	AmyA COG0366 505 GHs	Alpha-amylase_N|Alpha-amylase	AEH38729.1	GH13| CBM34
	LTtc-BRINE-C1834-cds9	beta-galactosidase	LacA COG1874 673 Beta-galactosidase	Glyco_hydro_42|Glyco_hydro_42M	AGB38905.1	GH42
	LTtc-BRINE-C3276-cds1	Levansucrase		Glyco_hydro_68	AGB39317.1	GH68
	LTtc-BRINE-C352-cds15	GH			AFZ71909.1	GH2
PL-Br10_E2g26	PL-BRINE-C17887-cds4	GH family 15	SGA1 COG3387 612 Glucoamylase and related GHs	Glyco_hydro_15	AFZ73398.1	GH15
	PL-BRINE-C9038-cds5	cyclomaltodextrinase	AmyA COG0366 505 GHs	Alpha-amylase_N|Alpha-amylase	AEH38729.1	GH13| CBM34
	PL-BRINE-C1577-cds16	GH			AFZ71909.1	GH2

Supplementary Table 12. Detected marker genes involved in carbon (C), nitrogen (N) and sulfur (S) metabolism from selected *Bacteroidetes*-related draft genomes from a selection of draft genomes. Marker genes were selected according to Lauro *et al*. (2011) and Llorens-Marès *et al*. (2015).

	Functional process	Knumber	Marker gene	T5-Br10_B2g0	T5-Br10_B2g13	Tc-Br11_B2g6_7
C	Anaerobic carbon fixation	K00174	2-oxoglutarate:ferredoxin oxidorecutase subunit alpha		+	+
		K00175	2-oxoglutarate:ferredoxin oxidoreductase subunit beta		+	+
	Fermentation	K00016	L-lactate dehydrogenase		+	+
	Aerobic respiration	K02274	cytochrome c oxidase subunit I (coxa)	+	+	+
		K02276	cytochrome c oxidase subunit III (coxc)	+	+	+
N	Nitrogen assimilation	K01915	glutamine syntetase (glnA)		+	+
	Mineralization	K00260	glutamate dehydrogenase		+	+
		K00261	glutamate dehydrogenase		+	+
		K00262	glutamate dehydrogenase		+	+
	Denitrification	K00376	nitrous oxide reductase (nosZ)		+	
S	Assimilatory sulfate reduction	K00957	sulfate adenylyltransferase subunit 2 (cysD)		+	
	Mineralization	K01011	3-mercaptopyruvate sulfurtransferase		+	

Supplementary Table 13. Putative glucoside hydrolases (GHs) identified in the gene repertoire from *Bacteroidetes*-related draft genomes. Predicted protein sequences were selected by sequence based annotation and Pfam rule based annotation using the CAZymes Analysis Toolkit (CAT; E-value 0.001, Bit-score 55). Query sequences were retained only if both their length and Pfam domains were consistent with the subject. Query Pfam domains and subject GenBank sequence identifiers (GI), as well as putative gene products of the besthits (Blast-N against; all e-values < 1E-05) and COG identifiers are given. CAZy families likely involved in polysaccharide degradation include chitinases (light green), cellulases and related glucanases (yellow), hemicellulases and related enzymes (dark red), enzymes acting on pectic polysaccharides (grey), glucoamylases and related GHs (orange). GH families likely involved in the hydrolysis of mono- and disaccharides are marked blue. Results classified under CAZy families GH 109 and GH 128 (encoding putative oxidoreductases and a RNA polymerase) respectively are not shown.

Draft	ContigID-cds#	Putative Gene product	COG	Pfam domains	Subject GI	CAZy Family
***Chitinophagaceae* bacterium T5-Br10_B2g13**	LT5-BRINE-C157-cds14	hypothetical protein	COG4623 473 Predicted soluble lytic transglycosylase fused to an ABC-type amino acid-binding protein	SBP_bac_3|SLT	AFU70457.1	GH23
	LT5-BRINE-C7091-cds7	hypothetical protein			AFJ21509.1	GH23
	LT5-BRINE-C8698-cds6	hypothetical protein			AFJ21509.1	GH23
	LT5-BRINE-C9827-cds10	hypothetical protein		Peptidase_M23	ABJ68049.1	GH23
	LT5-BRINE-C1119-cds5	hypothetical protein			AFN74880.1	GH51
	LT5-BRINE-C1965-cds2	hypothetical protein		DUF2233	AFN74880.1	GH51
	LT5-BRINE-C1965-cds5	hypothetical protein partial		DUF187	AFN74880.1	GH51
	LT5-BRINE-C2171-cds4	hypothetical protein		Glyco_hydro_3|Glyco_hydro_3_C|Fn3-like	AFH50282.1	GH3
	LT5-BRINE-C225-cds24	hypothetical protein			AFN74880.1	GH51
	LT5-BRINE-C3721-cds4	beta-glucosidase-like GH		Glyco_hydro_3|Glyco_hydro_3_C|Fn3-like	AFD06687.1	GH3
	LT5-BRINE-C6919-cds3	GH family 3	BglX COG1472 397 Beta-glucosidase-related GHs	Glyco_hydro_3	ACF12716.1	GH3
	LT5-BRINE-C84-cds10	hypothetical protein			AGC77489.1	GH17
	LT5-BRINE-C84-cds13	hypothetical protein	COG5309 305 Exo-beta-1,3-glucanase		AFU69871.1	GH17
	LT5-BRINE-C84-cds14	GH, partial			ACA88632.1	GH17
	LT5-BRINE-C84-cds8	hypothetical protein	SKN1 COG2273 355 Beta-glucanase/Beta-glucan synthetase	Glyco_hydro_16	AGC77483.1	GH16

Draft	ContigID-cds#	Putative Gene product	COG	Pfam domains	Subject GI	CAZy Family
***Chitinophagaceae* bacterium T5-Br10_B2g13 (continued)**	LT5-BRINE-C898-cds25	hypothetical protein AAW51_1390		Glyco_hydro_81	BAG20040.1	GH81
	LT5-BRINE-C3110-cds1	ABC transporter substrate-binding protein	XynB COG3507 549 Beta-xylosidase	Glyco_hydro_43	AEN72423.1	GH43
	LT5-BRINE-C84-cds11	hypothetical protein	COG5520 433 O-Glycosyl hydrolase	Glyco_hydro_30	EAQ41139.2	GH30
	LT5-BRINE-C2755-cds8	hypothetical protein			AFN74218.1	GH105
	LT5-BRINE-C179-cds17	4-alpha-glucanotransferase	MalQ COG1640 520 4-alpha-glucanotransferase	Glyco_hydro_77	AFZ29826.1	GH77
	LT5-BRINE-C185-cds22	hypothetical protein		Alpha-amylase	AFN75430.1	GH13
	LT5-BRINE-C18-cds4	glycosyltransferase 36		Glycoamylase|Glyco_transf_36|GT36_AF|CBM_X|Glyco_transf_36|GT36_AF	ADH61505.1	GH94|GT84
	LT5-BRINE-C2171-cds7	alpha amylase catalytic subunit		Alpha-amylase	AGC76529.1	GH13
	LT5-BRINE-C2182-cds4	alpha-amlyase		Alpha-amylase	AEE23801.1	GH13
	LT5-BRINE-C366-cds5	hypothetical protein	AmyA COG0366 505 GHs	Cyc-maltodext_N|Alpha-amylase|Cyc-maltodext_C	ADV49822.1	GH13
	LT5-BRINE-C6751-cds4	hypothetical protein			CBL12378.1	GH1
	LT5-BRINE-C691-cds6	hypothetical protein	LacZ COG3250 808 Beta-galactosidase/beta-glucuronidase	Glyco_hydro_2_N|Glyco_hydro_2|Glyco_hydro_2_C|Bgal_small_N	ABJ85566.1	GH2
	LT5-BRINE-C7036-cds3	alpha-mannosidase	COG3537 768 Putative alpha-1,2-mannosidase	Glyco_hydro_92	CBW20954.1 /CAH06071.1 /BAD47099.1	GH92
	LT5-BRINE-C418-cds24	trehalase tre37A	TreA COG1626 558 Neutral trehalase	Trehalase	ABS77572.1	GH37
	LT5-BRINE-C674-cds1	glucuronyl hydrolase		Glyco_hydro_88	ADY51417.1	GH88

Draft	ContigID-cds#	Putative Gene product	COG	Pfam domains	Subject GI	CAZy Family
***Flavobacteriaceae* bacterium T5-Br10_B2g0**	LT5-BRINE-C1669-cds7	lytic transglycosylase		SBP_bac_3|SLT	AFU70457.1	GH23
	LT5-BRINE-C5692-cds7	murein transglycosylase		SLT	AFU70471.1	GH23
	LT5-BRINE-C18102-cds1	hypothetical protein	LacZ COG3250 808 Beta-galactosidase/beta-glucuronidase	Glyco_hydro_16	AFU70112.1	GH16
	LT5-BRINE-C551-cds18	beta-N-acetylglucosaminidase		Glyco_hydro_3|Beta-lactamase	AFU70238.1	GH3
	LT5-BRINE-C6872-cds4	GH	COG4623 473 Predicted soluble lytic transglycosylase fused to an ABC-type amino acid-binding protein	Glyco_hydro_15	AFU68072.1	GH15
	LT5-BRINE-C918-cds1	hypothetical protein		Glyco_hydro_2_N|Glyco_hydro_2|Glyco_hydro_2_C|Bgal_small_N	ABG39349.1/ AEE24439.1	GH2
	LT5-BRINE-C930-cds10	GH	SGA1 COG3387 612 Glucoamylase and related GHs	Glyco_hydro_2_N|Glyco_hydro_2|Glyco_hydro_2_C	CAZ97771.1	GH2
***Rhodothermaceae* bacterium Tc-Br11_B2g6_7**	LTtc-BRINE-C22-cds36	GH family 3, partial		Glyco_hydro_3|Glyco_hydro_3_C|Fn3-like	CBH25982.1	GH3
	LTtc-BRINE-C247-cds20	hypothetical protein partial		Glyco_hydro_3	AFY75765.1	GH3
	LTtc-BRINE-C667-cds2	hypothetical protein			AFN74927.1	GH5|CBM35
	LTtc-BRINE-C48-cds44	hypothetical protein		GTA_TIM	ABC45350.1	GH113
	LTtc-BRINE-C130-cds11	glycosyl hydrolase family 15	SGA1 COG3387 612 Glucoamylase and related GHs	Glyco_hydro_15	ACV68191.1	GH15
	LTtc-BRINE-C2009-cds2	hypothetical protein	AmyA COG0366 505 GHs	Alpha-amylase	AFL85845.1	GH13
	LTtc-BRINE-C3-cds14	4-alpha-glucanotransferase	MalQ COG1640 520 4-alpha-glucanotransferase	Glyco_hydro_77	ACY47896.1	GH77
	LTtc-BRINE-C3-cds7	trehalose synthase		Alpha-amylase	ABY34390.1/ ACM52692.1	GH13
	LTtc-BRINE-C3-cds8	alpha-1,4-glucan:maltose-1-phosphate maltosyltransferase		DUF3416	ADJ29108.1	GH13
	LTtc-BRINE-C48-cds20	alpha-amylase		Alpha-amylase	AEN73683.1	GH13
	LTtc-BRINE-C48-cds6	fructofuranosidase/invertase		n/a	AGA90774.1	GH100
